# CoRa—A general approach for quantifying biological feedback control

**DOI:** 10.1073/pnas.2206825119

**Published:** 2022-08-29

**Authors:** Mariana Gómez-Schiavon, Hana El-Samad

**Affiliations:** ^a^Department of Biochemistry and Biophysics, University of California, San Francisco, CA 94158;; ^b^Laboratorio Internacional de Investigación sobre el Genoma Humano, Universidad Nacional Autónoma de México, Santiago de Querétaro 76230, México;; ^c^Millennium Science Initiative Program, Millennium Institute for Integrative Biology (iBio), Chilean National Agency for Research and Development, Santiago 8331150, Chile;; ^d^Cell Design Initiative, University of California, San Francisco, CA 94158;; ^e^Chan–Zuckerberg Biohub, San Francisco, CA 94158;; ^f^Cell Design Institute, University of California, San Francisco, CA 94158

**Keywords:** feedback, homeostasis, control

## Abstract

Feedback control is a fundamental underpinning of life, underlying homeostasis of biological processes at every scale of organization, from cells to ecosystems. The ability to evaluate the contribution and limitations of feedback control mechanisms operating in cells is a critical step for understanding and ultimately designing feedback control systems with biological molecules. Here, we introduce CoRa—or Control Ratio—a general framework that quantifies the contribution of a biological feedback control mechanism to adaptation using a mathematically controlled comparison to an identical system that does not contain the feedback. CoRa provides a simple and intuitive metric with broad applicability to biological feedback systems.

Feedback control is a mechanism by which a system can assess its own state and use this information to react accordingly (1). Cells and organisms make abundant use of feedback control (2), in particular, negative feedback to deploy corrective actions. Negative feedback is instrumental in the ability of biological systems to restore homeostasis after a perturbation (3–7), a property known, in engineering, as disturbance rejection and, in the biological sciences, as adaptation. Despite the importance of feedback, no systematic and generalizable approaches exist to quantify the contribution of a negative feedback loop to adaptation in biological networks. Here, we propose CoRa—or Control Ratio—a mathematical approach that tackles this problem. CoRa follows the classical notion of mathematically controlled comparisons (8) by assessing the performance of a biological system with feedback control to a locally analogous system without feedback. The locally analogous system without feedback has identical structure and parameters to those of the feedback system, except for the feedback link, and both systems rest at the same steady-state value before the perturbation. As a result, the divergence in their behavior after they are challenged with a perturbation isolates and quantifies the contribution of the feedback control (Fig. 1). The fundamentals behind this approach were previously developed by Alves and Savageau (8–10) under a strict mathematical formalism, proposing the feedback effectiveness metric (*SI Appendix*). CoRa can be defined and computed for any biological system described by a solvable set of ordinary differential equations, irrespective of its complexity. CoRa can also be efficiently computed across different parameter values of a system, allowing a global view of the performance of its feedback under different conditions.

**Fig. 1.**
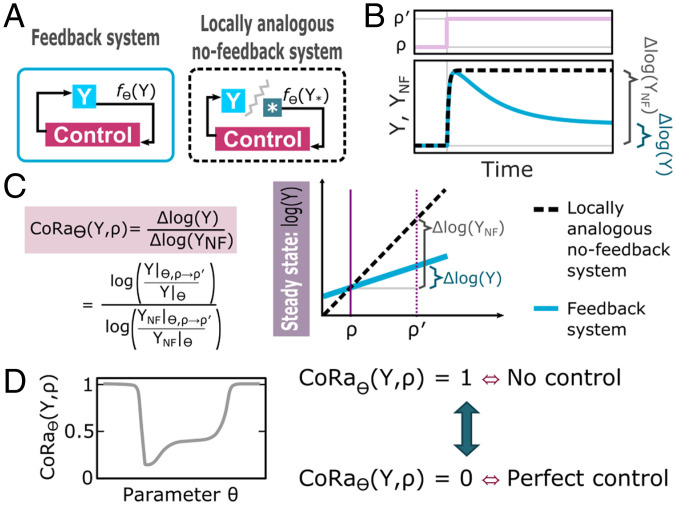
Explaining the CoRa approach. (*A*) Diagram of a system with feedback (*Left*) and its locally analogous system without feedback (*Right*). Here, *Y* corresponds to the controlled species on which to evaluate the effect of the feedback control (*_NF_* subindex to denote the system without feedback). For a given parameter set (Θ), the constant input is fixed such that the feedback signal $\left(f_{\Theta }(Y)\right)$ remains analogous to the system without feedback before any perturbation is applied to both systems. (*B*) Plot of the response of the system with feedback (blue line) and the locally analogous system (black dashed line) as functions of time after a small perturbation to a specific parameter (ρ→ρ′, with ρ∈Θ). (*C*) Definition of CoRa. For each parameter set Θ, the CoRa value for perturbation to *ρ*, ${\text{CoRa}}_{\Theta }(Y,\rho )$, is defined as the ratio of *Y* change of the feedback (Δlog(Y)) and no-feedback locally analogous $\left(\Delta \text{log}(Y_{NF})\right)$ systems after a small perturbation (ρ→ρ′). *Left* gives the formula for CoRa and *Right* shows a graphical interpretation of this quantity. (*D*) ${\text{CoRa}}_{\Theta }(Y,\rho )$ for perturbations in *ρ* can be calculated across a range of values of a chosen parameter θ∈Θ.

## CoRa Formulation

To apply CoRa, two systems are considered: the intact system that has the feedback structure, and a locally analogous system without feedback, each of them described by a set of ordinary differential equations with parameters Θ. The locally analogous system is designed to have exactly the same biochemical reactions as the feedback system, with the only difference being the removal of the direct influence of the controlled species (*Y*) over the rest of the system, therefore generating a system without feedback. Instead, a constant input is introduced in the locally analogous system that mimics the direct influence of *Y* on the relevant chemical species in the system. This positions both systems, the feedback and its locally analogous system with no feedback, at identical steady-state values for the controlled species and all internal variables under the given parameter set Θ. A step-by-step procedure for generating such an analogous system is detailed in *SI Appendix*.

Once the feedback system and locally analogous system without feedback are defined in this way, the broad idea of CoRa is that, in order to evaluate the contribution of the feedback to adaptation of a species *Y* following a perturbation in a specific parameter ρ∈Θ, one can apply a small perturbation ($\rho \to \rho^{\prime }$) and compare the response of the two systems. More specifically, the contribution of the feedback to adaptation of *Y* after a perturbation in parameter ρ∈Θ can be quantified as the ratio of the response of the feedback system (Δlog(Y)) and its locally analogous system without feedback (denoted with the subindex *_NF_*; $\Delta \text{log}(Y_{NF})),\;{\text{CoRa}}_{\Theta }(Y,\rho )=\Delta \text{log}(Y)/\Delta \text{log}(Y_{NF})$ (Fig. 1*C*). Being locally analogous, the two compared systems possess the same nonlinearities and saturations under the given parameter set Θ. As a result, any differences in the response to a small perturbation are attributed to the effect of feedback (*SI Appendix*).

CoRa provides an easily interpretable assessment of how a system with feedback, positioned at the parameter set Θ, fares compared to a no-feedback system when *ρ* is perturbed. For instance, if ${\text{CoRa}}_{\Theta }(Y,\rho )\in [0,1)$, the presence of the feedback reduces the effect of the perturbation compared to the locally analogous system without feedback, $\Delta \text{log}(Y)<\Delta \text{log}(Y_{NF})$ (*SI Appendix*). When ${\text{CoRa}}_{\Theta }(Y,\rho )=0$, the feedback endows the system with perfect adaptation (Δlog(Y)=0), with the controlled species returning exactly to the preperturbed state even in the continued presence of the perturbation. The value of ${\text{CoRa}}_{\Theta }(Y,\rho )$ increases as the control effect decreases, and when ${\text{CoRa}}_{\Theta }(Y,\rho )=1$, the feedback is ineffective, as the controlled species of the system with feedback becomes indistinguishable from that of the system without feedback (i.e., $\Delta \text{log}(Y)=\Delta \text{log}(Y_{NF})$). This procedure can be repeated for any parameter set of interest. Specifically, we can compute CoRa for a range of values of any parameter in Θ (*SI Appendix*) while adjusting the constant input of the no-feedback system (as explained above) accordingly to ensure the mathematically controlled comparison in the sense we describe above.

### Using CoRa to Characterize Negative Feedback in a System Architecture Capable of Perfect Adaptation.

**Fig. 2.**
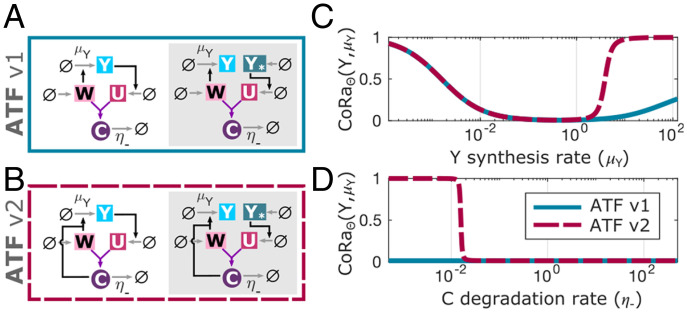
Characterizing the antithetic feedback motif (ATF) using CoRa. The ATF motif is composed by two molecules (*W*, *U*) that bind and inactivate each other, forming a transitory complex *C* which is then degraded with rate $\eta_{-}$; one antithetic molecule *W* induces *Y* synthesis (the controlled species, with rate *μ_Y) _*, and *Y* feeds back by inducing the synthesis of the other antithetic molecule, *U*. We consider two variations of the feedback structure. (*A*) The first (ATF v1; blue continuous lines) is akin to the original ATF motif, with the difference that the binding of *U* and *W* generates a complex *C* that is explicitly modeled before it disappears through degradation at a rate $\eta_{-}$. (*B*) In the second feedback structure (ATF v2; pink dash lines), the complex *C* retains biological activity in influencing the production of *Y* until it is degraded. This structure is inspired by the feedback implementation documented in Ng et al. (12). For each case, the associated locally analogous system without feedback (shaded area) is shown. (*C*) CoRa computed following perturbations to *μ_Y, _* the synthesis rate of *Y*, as this parameter itself is varied. (*D*) CoRa computed following a perturbation to *μ_Y _*as the degradation rate of the complex $C(\eta_{-})$ is varied. See *SI Appendix* for equations and parameter values.

We tested CoRa on a well-established negative feedback control structure, the antithetic feedback motif, which can exhibit perfect adaptation to step disturbances when connected to an arbitrarily complex biochemical network (11) (Fig. 2). The antithetic motif is composed of two molecular species that annihilate each other through their mutual binding. One of the antithetic molecular species controls the input of a biochemical network, and the other is produced by the output of the same network. If the antithetic molecules are only lost through the mutual annihilation event without individual degradation or dilution, this strategy is expected to generate a system with perfect adaptation to a step perturbation (11). Using CoRa to study this feedback motif, we recapitulate this result, showing that perfect adaptation is possible (Fig. 2). Interestingly, our analysis also reveals that relaxing the assumption of zero dilution and adding molecular details such as explicit accounting of the transitory molecule resulting from binding of the two antithetic molecules (complex *C* in Fig. 2*A*) is sufficient to compromise perfect adaptation, often in nontrivial ways (Fig. 2 *C* and *D*). For example, ${\text{CoRa}}_{\Theta }(Y,\mu_{Y})$ (*μ_Y _*is the synthesis rate of the controlled species *Y*) deviates from perfect adaptation value of zero if dilution of antithetic molecules is assumed to occur individually at a small rate $\gamma =10^{-4}\;{\text{min}}^{-1}$. This deviation from perfect adaptation occurs at low and high values of *μ_Y _*(Fig. 2*C* and *SI Appendix*). In a further elaboration of the circuit, when we consider the complex *C* as a functional molecule that can influence the synthesis of the controlled molecule *Y* until its removal from the system (12) (Fig. 2*B*), the feedback undergoes a dramatic failure in its ability to produce perfect adaptation after a specific threshold value of *μ_Y _*. This is evidenced by ${\text{CoRa}}_{\Theta }(Y,\mu_{Y})$ shifting abruptly from almost zero to one (Fig. 2*C*). This is also the case when lowering the degradation rate of complex *C*, $\eta_{-}$ (Fig. 2*D*). Exploration of this phenomenology identified by CoRa reveals that this qualitative change in the feedback control results from saturation in the concentration of the complex *C* (*SI Appendix*), an insight that would have been difficult without the computational observation of this behavior.

### Using CoRa to Compare Different Feedback Control Mechanisms.

**Fig. 3.**
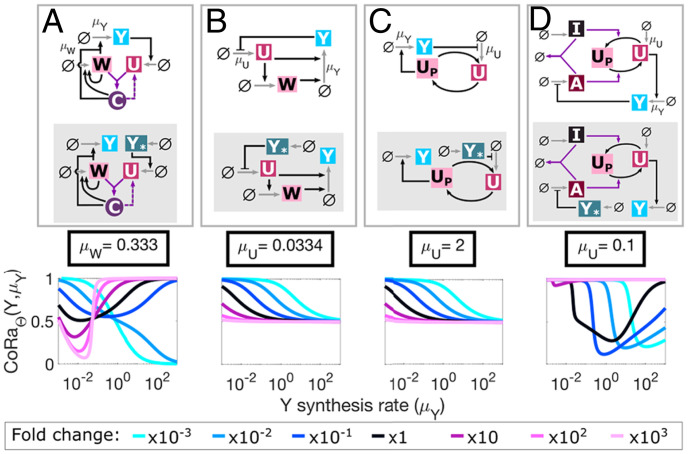
CoRa provides a unifying framework to compare different feedback control architectures. (*A*–*D*) Different feedback motifs, with different levels of complexity, can be directly compared using the CoRa function. In each case, the diagrams of the feedback system and its associated locally analogous system without feedback (shaded area) are shown. ${\text{CoRa}}_{\Theta }(Y,\mu_{Y})$ is computed for seven different values of a given parameter that is also varied in addition to *μ_Y _*. The identity and nominal value of the varied parameter (either *μ_W_*, the *W* synthesis rate, or *μ_U_*, the *U* synthesis rate) is indicated on every plot (black line and box), and how it is varied is shown at the fold change legend. See *SI Appendix* for equations and parameter values.

Any feedback control system can be analyzed using CoRa, providing a unifying framework under which different feedback mechanisms can be rigorously compared. As an example, we compared four structurally diverse feedback control motifs (13–16) (Fig. 3 and *SI Appendix*). We observed that the feedback strategies employing repression of synthesis modeled using a standard Michaelis–Menten repression function had a limit of ${\text{CoRa}}_{\Theta }(Y,\mu_{Y})\geq 0.5$ (Fig. 3 *B* and *C*), except when ultrasensitivity was present (Fig. 3*D*). This prompted the hypothesis that Michaelian repression severely limits homeostatic capacity (as the function saturates), but this can be alleviated using ultrasensitive components. Using CoRa, we tested this hypothesis and confirmed that increasing the system ultrasensitivity (e.g., Hill coefficient larger than one) decreased the lower bound of CoRa values (*SI Appendix*). This observation is in agreement with previous observations (10). Here, CoRa was used as a computational hypothesis generator about this general principle, which was then confirmed through further computational and analytical investigations (*SI Appendix*).

### Discussion.

A framework for the systematic evaluation and comparison of biochemical feedback control systems is essential for understanding the general principles of biological homeostasis. While many methods exist for the evaluation of technological feedback systems, understanding the principles of biological adaptation mediated through feedback poses its unique challenges, including distinct mathematical properties of the biological substrate. Importantly, the nature of biological organization with extensive coupling of parameters and processes makes the extraction of engineering-centric quantities needed for traditional analyses of feedback quantities, such as setpoints and regulation errors, challenging. Debate about whether these quantities are defined for biological systems has a long history and no concrete resolution (17). For example, a system displaying ultrasensitivity or biochemical saturation—ubiquitous properties of biological systems, for example, from enzymatic reactions to transcriptional regulation of promoters with multiple binding sites—can wrongly be described as having feedback control with approximately “zero error,” given that these properties exhibit negligible change in the controlled species for a range of conditions. Nevertheless, the underlying mechanisms and implications of this lack of response are very different from feedback control. One advantage of CoRa is that it does not make any assumptions about the existence of such quantities, replacing this debate with a comparison to a system that would have evolved identically but without the feedback structure. Another advantage of CoRa is that it is agnostic to the complexity of the system. While we have only used simple systems to illustrate the properties of CoRa, extending analysis to more complex systems is straightforward. We have also used CoRa to assess feedback-mediated adaptation for only one perturbation as a function of one model parameter. However, it should be easy to see that a multidimensional CoRa for simultaneous perturbations or many concurrent parameter changes is easily computable. We expect, however, that new methods would be needed to analyze the resulting multidimensional data into coherent principles.

Evidently, constructing the locally analogous system without feedback for a case of interest can be challenging in an experimental setting. We propose that, by testing multiple constructions for a range of conditions (e.g., using different constitutive promoters (12)), it is possible to find conditions where the controlled species of the systems with feedback and that without feedback coincide, therefore allowing for experimental determination of CoRa.

Finally, the concept of CoRa should be easily extendable to assessing the quantitative contribution of feedback to important properties other than the steady-state response to perturbation. These might, for example, include the role of feedback in the dynamic response of a system or its response to stochastic fluctuations. As such, CoRa represents a flexible framework that is poised to catalyze fast progress in our understanding of the many roles that feedback control plays in biological organization.

## Supplementary Material

Supplementary File

## Data Availability

Code and simulations have been deposited in GitHub (https://github.com/mgschiavon/CoRa), and Zenodo (doi: 10.5281/zenodo.5842431) (18, 19). Equations, parameters values, and algorithms also appear in *SI Appendix*.
