# Supplementary material for: CoRa—A general approach for quantifying biological feedback control
Source: Proc Natl Acad Sci U S A. 2022 Aug 29;119(36):e2206825119. doi: 10.1073/pnas.2206825119 (PMC9457221; doi:10.1073/pnas.2206825119)
Supplement: Supplementary File [file pnas.2206825119.sapp.pdf]

SI Appendix

CoRa –A general approach for quantifying biological feedback control

Mariana Gómez-Schiavon[1,5,6,*], Hana El-Samad[1,2,3,4,*]

1 Department of Biochemistry and Biophysics, University of California, San Francisco, San Francisco, CA, USA.
2 Cell Design Initiative, University of California, San Francisco, CA, USA
3 Chan–Zuckerberg Biohub, San Francisco, CA, USA.
4 Cell Design Institute, San Francisco, CA, USA.
5 Laboratorio Internacional de Investigación sobre el Genoma Humano, Universidad Nacional Autónoma de México, Santiago de Querétaro, México.
6 ANID—Millennium Science Initiative Program—Millennium Institute for Integrative Biology (iBio), Santiago 8331150, Chile.

*** MGSchiavon@liigh.unam.mx; Hana.El-Samad@ucsf.edu**

Contents

S1 Foundations **3**

S2 CoRa approach **3**
S2.1 Steps for CoRa implementation 5
S2.2 Algorithm for CoRa implementation 7

S3 Analysis of a modified antithetic feedback control strategy using CoRa **7**
S3.1 Understanding effect of saturation on modified antithetic feedback control 10
S3.1.1 ATF control limits with inactive complex 10
S3.1.2 ATF control limits with active complex 13

S4 Negative auto-regulation affecting synthesis represented by Michaelis-Menten function limits control performance in multiple motifs, but is alleviated by ultrasensitivity. **15**
S4.1 Understanding effect of saturation on buffering + negative feedback control strategy 15
S4.2 *Feedback effectiveness* metric, control saturation and ultrasensitivity 18

S5 Comparing Feedback Control Motifs with CoRa **18**
S5.1 Feedback by Active Degradation + Positive Feedback with inactive complex 18
S5.2 Feedback + Feedforward Loop 19
S5.3 Buffering + Negative Feedback 19
S5.4 Brink Motif Feedback 20

S6 Parameter values and models used in the manuscript figures **21**

Note: Across this document, capital letters (e.g. X) represent both the species and its concentration; the sub-index X_{ss} refers to the steady state value; and lower-case Greek letters represent parameters, which by default are non-negative real numbers.

S1 Foundations

The main conceptual ingredients of the CoRa approach have been developed by Savageau and colleagues a number of decades ago. First, they showed how the **local relative parameter sensitivity** can be used to quantitatively measure the properties of organized networks of enzyme-catalyzed reactions [11]. The local relative parameter sensitivity measures the relative variation on some property of interest in a specific system in steady state (e.g. control) with respect to the relative variation of a chosen parameter (e.g. the parameter representing the perturbation). Building on this idea, in a second publication on 1971, they used the **ratio of sensitivities** involving a controlled and an otherwise equivalent uncontrolled system to evaluate the effect of feedback control by inhibition on the end-product of a biosynthetic pathway, proposing a metric called *feedback effectiveness* (FE) [10] . In this paper, Savageau applied a formal approximation to simplify the studied system, allowing an analytical treatment. Under this approximation, kinetic models are rewritten to a general canonical nonlinear form, known as S-systems (see [12]), following the Power-Law formalism. Briefly, under this S-system form, each nonlinear differential equation of the model is written aggregating all processes that contribute to the increase and to the decrease of the species in power-law functions, $\frac{d}{dt}X_i = \alpha_i \Pi_{j=0}^{n} X_j^{g_{i,j}} - \beta_i \Pi_{j=0}^{n} X_j^{h_{i,j}}$, where $g_{i,j}, h_{i,j}$ are the "apparent kinetic orders" of the rate terms of species X_j with respect to the species X_i, and $\alpha_i, \beta_i \in \mathbb{R}_{0+}$ (see [12] for more details). Using this S-system model, Savageau [10] defines first the *logarithmic gain* of the system output species as the input species change (L), which is shown to depend only on the g and h structural parameters of the system (i.e. independent of the particular concentration value of the species). Then, they calculated the sensitivity of this logarithmic gain to changes on one of these structural parameters (e.g. $S_{L,h} = \frac{\partial L}{\partial h}\frac{h}{L}$), and evaluated the ratio of sensitivity of the system with ($S_{L,h}, S_{L,g}$) and without feedback ($S'_{L,h}, S'_{L,g}$). The analytical treatment of the S-system abstraction allows them to define a simple expression for this ratio, and they defined the *feedback effectiveness* (FE) as $FE = \big(1 - \frac{S_{L,h}}{S'_{L,h}}\big)/\big(\frac{S_{L,h}}{S'_{L,h}}\big) = \big(1 - \frac{S_{L,g}}{S'_{L,g}}\big)/\big(\frac{S_{L,g}}{S'_{L,g}}\big)$. FE represents then a quantitative measurement of the potency of the feedback, with FE equal zero when there is no feedback, and increases its value as the strength of the feedback increases. In this particular case, this FE metric is related to the CoRa metric developed here (see Section S2), with $CoRa = 1/(1 + FE)$.

Later, Savageau developed a generalization of the comparison between two systems that the differ only by one element, coined it as **mathematically controlled comparison** [9]. This technique allows us to determine irreducible differences in the response of the two compared systems (see Section S2 for a wider description in the context of CoRa). And more recently, Savageau and colleagues (2000) [1] expanded the application of mathematically controlled comparisons to a numerical implementation. Furthermore, Savageau and colleagues have shown the power of the mathematically controlled comparison through multiple applications, including the evaluation of robustness of synthetic oscillators [6], and for metabolic engineering strategies [13].

The parallelisms between CoRa and FE are validating for both of them (e.g. see Section S4.2). The FE metric has the advantage of being supported on a strict mathematical formalism, which can be approached analytically. Nevertheless, this requires recasting any kinetic model into an S-system formalism [12]. This can be cumbersome, and as we demonstrate here, unnecessary to apply CoRa. As a result, CoRa brings a new perspective to the issue of quantifying feedback control from a synthetic biology point of view, making this quantification facile, and making it more approachable for the biology community in general.

S2 CoRa approach

CoRa –or ***Co**ntrol **Ra**tio*– aims to quantify the effect of feedback control on a system's ability to reject a step perturbation, while considering the effect and constraints of the individual biochemical events. This is done by directly comparing the feedback system of interest to a locally analogous system without feedback under the formalism of *mathematically controlled comparisons* [1]. Each locally analogous system has exactly the same biochemical reactions and parameters as the original feedback system (i.e. *internal equivalence*), with the exception

of the feedback link from the controlled subsystem. For each specific parameter set Θ (i.e. the value of all parameters describing the system of interest), the feedback link is substituted by an equivalent constant input calibrated such that the steady-state of all common species between the two systems are identical before a perturbation is applied (i.e. *external equivalence*). This equivalence allows for a direct comparison of the controlled species change of both systems following a specific step perturbation (e.g. step change in a parameter value), while accounting for the influence of the nonlinearity, saturation, and other intrinsic particularities of the system, and guarantying that any differential response of these two analogous systems represents an *inherent functional difference* associated with the feedback control. The perturbation considered must not affect the constant input of the locally analogous system, as otherwise the differential response can no longer be uniquely associated with the feedback control.

Let Y be the species over which we want to evaluate the feedback control effect, $Y_{ss}|_\Theta$ denote the steady-state value of the system with feedback for a parameter set Θ, and $Y_{ss,NF}|_\Theta$ denote the steady-state value of the locally analogous system without feedback. Let's also consider a small step perturbation of a specific parameter $\rho \in \Theta$ ($\rho \to \rho'$). Following this perturbation, $Y_{ss}|_{\Theta,\rho\to\rho'}$ and $Y_{ss,NF}|_{\Theta,\rho\to\rho'}$ denote that new steady-states of the feedback system and locally analogous system without feedback, respectively.

CoRa is then defined as:

$$\begin{aligned}\mathrm{CoRa}_\Theta(Y,\rho) &= \frac{\Delta\log(\mathrm{Y_{ss}})|_{\Theta,\rho\to\rho'}}{\Delta\log(\mathrm{Y_{ss,NF}})|_{\Theta,\rho\to\rho'}} \qquad \text{(S1)}\\ &= \frac{\log(Y_{ss}|_{\Theta,\rho\to\rho'}) - \log(Y_{ss}|_\Theta)}{\log(Y_{ss,NF}|_{\Theta,\rho\to\rho'}) - \log(Y_{ss,NF}|_\Theta)}\\ &= \frac{\log\left(\frac{Y_{ss}|_{\Theta,\rho\to\rho'}}{Y_{ss}|_\Theta}\right)}{\log\left(\frac{Y_{ss,NF}|_{\Theta,\rho\to\rho'}}{Y_{ss,NF}|_\Theta}\right)}\end{aligned}$$

Note that by construction the steady state value of the controlled species in the feedback system and the locally analogous system without feedback are identical before a perturbation, i.e. $Y_{ss}|_\Theta = Y_{ss,NF}|_\Theta$.

Assuming that $\Delta\rho = \rho' - \rho$ is small enough, the steady state value of the controlled species in the feedback system and the locally analogous system without feedback can be expressed as linear functions of $\Delta\rho$. The corresponding CoRa function can then be written as:

$$\begin{aligned}\mathrm{CoRa}_\Theta(Y,\rho) &= \frac{\log(Y_{ss}(\rho+\Delta\rho)) - \log(Y_{ss}(\rho))}{\log(Y_{ss,NF}(\rho+\Delta\rho)) - \log(Y_{ss,NF}(\rho))}\\ &\approx \frac{\log(Y_{ss}(\rho)) + \Delta\rho\frac{d}{d\rho}\log(Y_{ss})|_\rho - \log(Y_{ss}(\rho))}{\log(Y_{ss,NF}(\rho)) + \Delta\rho\frac{d}{d\rho}\log(Y_{ss,NF})|_\rho - \log(Y_{ss,NF}(\rho))}\\ &\approx \frac{\frac{d}{d\rho}\log(Y_{ss})|_\rho}{\frac{d}{d\rho}\log(Y_{ss,NF})|_\rho} \qquad \text{(S2)}\end{aligned}$$

Eq. S2 shows that in this regime, CoRa value is approximately independent of the perturbation size $\Delta\rho$. In all the analyses presented on this paper, we used $\rho' = 1.05\rho$. We corroborated that this perturbation size was small enough to reach the linear regime by confirming that identical results were obtained with $\rho' = 1.01\rho$. Nevertheless, with the smaller perturbation size ($\rho' = 1.01\rho$), noise in the numerical solutions was observed for some cases. In general, like for any linearization exercise, the acceptable perturbation size for numerical solutions needs to be evaluated for the specific system and conditions of interest.

The value of $\mathrm{CoRa}_\Theta(Y,\rho)$ can be easily related to the logic of the feedback. We can abstract the control system to a two-node network where one node represents the controlled species (Y), and the other the rest of the system including the dependency on the parameter ρ to be perturbed ($x(\rho)$). The locally analogous system can be represented as an equivalent network, with a third node ($*$) that represent the new input into the $x(\rho)$ node. The other link from $x(\rho)$ to the controlled species (Y_{NF}; link #1) remains the same between the two networks. The sign of link #1 can be determined by comparing the controlled species before ($Y_{NF}|_\Theta = Y|_\Theta$) and after ($Y_{NF}|_{\Theta,\rho\to\rho'}$) the perturbation in the locally analogous system without feedback. For a positive perturbation, link #1 is positive (#1 (+)) if and only if $Y_{NF}|_{\Theta,\rho\to\rho'} > Y|_\Theta$, or negative (#1 (−)) if and only if $Y_{NF}|_{\Theta,\rho\to\rho'} < Y|_\Theta$. The sign of the feedback link from the controlled species to the $x(\rho)$ node (link #2) can be determined by comparing the controlled

species after the perturbation in the feedback system ($Y|_{\Theta,\rho\to\rho'}$) and in the locally analogous system ($Y_{NF}|_{\Theta,\rho\to\rho'}$). It is positive (#2 (+)) if and only if $Y|_{\Theta,\rho\to\rho'} > Y_{NF}|_{\Theta,\rho\to\rho'}$, or negative (#2 (−)) if and only if $Y|_{\Theta,\rho\to\rho'} < Y_{NF}|_{\Theta,\rho\to\rho'}$. Given the formula for CoRa (Eq. S1), we can see that $\text{CoRa}_\Theta(Y,\rho)$ is bound between 0 and 1 whenever we have a negative feedback, and bigger than 1 in the case of a positive feedback.

Then, if $\text{CoRa}_\Theta(Y,\rho) \in [0,1)$, the presence of the feedback reduces the effect of the perturbation compared to the locally analogous system without feedback, i.e. the system has an active negative feedback: either $0 \leq \Delta\log(Y_{ss})|_{\Theta,\rho\to\rho'} < \Delta\log(Y_{ss,NF})|_{\Theta,\rho\to\rho'}$ or $0 \geq \Delta\log(Y_{ss})|_{\Theta,\rho\to\rho'} > \Delta\log(Y_{ss,NF})|_{\Theta,\rho\to\rho'}$. On the other hand, if $\text{CoRa}_\Theta(Y,\rho) > 1$, the presence of the feedback amplifies the effect of the perturbation compared to the locally analogous system without feedback, i.e. the system has an active positive feedback: either $\Delta\log(Y_{ss})|_{\Theta,\rho\to\rho'} > \Delta\log(Y_{ss,NF})|_{\Theta,\rho\to\rho'} > 0$ or $\Delta\log(Y_{ss})|_{\Theta,\rho\to\rho'} < \Delta\log(Y_{ss,NF})|_{\Theta,\rho\to\rho'} < 0$. Finally, if $\text{CoRa}_\Theta(Y,\rho) = 1$, the feedback is effectively inactive. As the goal of CoRa is to quantify feedback control, which by definition requires a corrective (negative) feedback regulation, $\text{CoRa}_\Theta(Y,\rho)$ is bounded between 0 and 1 for the cases of interest. More specifically, $\text{CoRa}_\Theta(Y,\rho) = 0$ only if the system displays perfect control ($Y_{ss}|_{\Theta,\rho\to\rho'} = Y_{ss}|_\Theta$), and $\text{CoRa}_\Theta(Y,\rho)$ value increases as the control effect decreases up until $\text{CoRa}_\Theta(Y,\rho) = 1$, when the feedback contribution is effectively zero (i.e. the system response to the perturbation is exactly the same that the one of the system without feedback).

S2.1 Steps for CoRa implementation

1. Define a solvable **set of ordinary differential equations** representing the biological system of interest, where each equation describes the dynamics of the concentration of a molecular species involved in the system. For example (see Section S3 for the full description of the biological system associated with these equations):

$$
\begin{aligned}
\frac{d}{dt}U &= \mu_U Y - (\gamma+\gamma_U)U - \eta_+ UW + (\eta_0+\gamma_W)C \\
\frac{d}{dt}W &= \mu_W - (\gamma+\gamma_W)W - \eta_+ UW + (\eta_0+\gamma_U)C \\
\frac{d}{dt}C &= \eta_+ UW - (\gamma+\eta_0+\eta_- +\gamma_U+\gamma_W)C \\
\frac{d}{dt}Y &= \mu_Y W - (\gamma+\gamma_Y)Y
\end{aligned}
$$

2. Define the **controlled species** of interest over which to evaluate the effect of the feedback control; the analysis can be repeated for diverse species in the system. In the differential equations example above, a controlled species of interest can be Y, and we may be particularly interested in its steady-state value.

3. Determine the **feedback link functions** as all functions dependent on the controlled species defined above (e.g. Y) through which this species influences the other molecular species (e.g. U, W, C). Feedback link functions are therefore the links from the defined controlled subsystem to the rest of the system. In the example described above, the unique feedback link function ($f_\theta(Y)$) is the regulated synthesis function of U dependent on Y:

$$f_\Theta(Y) = \mu_U Y$$

   Note: In general, CoRa can be calculated using a downstream element representing a measurement of the controlled species of interest, e.g. a reporter protein. Nevertheless, when determining the feedback functions, the directly controlled species –which is part of the feedback loop– must be considered.

4. Build the **locally analogous no-feedback system** as an identical set of equations as the full, feedback-controlled system, except that the feedback link functions are substituted by constant inputs. These inputs are not dependent on the controlled species (e.g. Y), but have identical magnitudes when evaluated in the pre-perturbation steady-state for the given condition (i.e. Θ). We can accomplish this through two alternative strategies:

   (a) Introduce some auxiliary species with constitutive expression (i.e. not regulated by any other molecule in our system) with a pre-perturbation steady-state concentration that matches the concentration of the

regulatory species in the feedback link functions, i.e. the controlled species. Then, use the auxiliary species in the feedback link functions. Using this strategy, the locally analogous no-feedback system for the example described above would be:

$$\begin{aligned}
\frac{d}{dt}U &= \mu_U Y_* - (\gamma + \gamma_U)U - \eta_+ UW + (\eta_0 + \gamma_W)C \\
\frac{d}{dt}W &= \mu_W - (\gamma + \gamma_W)W - \eta_+ UW + (\eta_0 + \gamma_U)C \\
\frac{d}{dt}C &= \eta_+ UW - (\gamma + \eta_0 + \eta_- + \gamma_U + \gamma_W)C \\
\frac{d}{dt}Y &= \mu_Y W - (\gamma + \gamma_Y) \\
\frac{d}{dt}Y_* &= \mu_{Y_*} - (\gamma + \gamma_{Y_*})Y_*
\end{aligned}$$

where Y_* represents the auxiliary (non-regulated) species, which is constitutively expressed with synthesis $\mu_{Y*} = \mu_Y W_{ss}$, and degradation $\gamma_{Y*} = \gamma_Y$, such that the steady-state value of the locally analogous system without feedback $Y_{ss,NF}$ is equal to the steady state value of the feedback system Y_{ss} before a perturbation to both systems takes them away from that identical steady-state.

(b) Substitute the feedback link functions dependent on the controlled species (e.g. $f_\Theta(Y) = \mu_U Y$ in the example above) with a constant whose value is identical to the feedback link function values evaluated at the pre-perturbation steady-state. Using this strategy, the locally analogous no-feedback system for the example described above would be:

$$\begin{aligned}
\frac{d}{dt}U &= \mu_{U*} - (\gamma + \gamma_U)U - \eta_+ UW + (\eta_0 + \gamma_W)C \\
\frac{d}{dt}W &= \mu_W - (\gamma + \gamma_W)W - \eta_+ UW + (\eta_0 + \gamma_U)C \\
\frac{d}{dt}C &= \eta_+ UW - (\gamma + \eta_0 + \eta_- + \gamma_U + \gamma_W)C \\
\frac{d}{dt}Y &= \mu_Y W - (\gamma + \gamma_Y)Y
\end{aligned}$$

where U is now constitutively expressed with synthesis $\mu_{U*} = \mu_U Y_{ss}$, such that here again, the steady state value of the locally analogous system without feedback $Y_{ss,NF}$ is equal to the steady state value of the feedback system Y_{ss}.

The goal here is that both systems (the original feedback system and its locally analogous system without feedback) have not only identical steady-state values for all species in the condition being evaluated, but if a perturbation occurs, both systems would respond initially in an identical manner, as all the links (regulatory functions) transmit exactly the same information (e.g. with identical levels of nonlinearity and saturation), with the clear and intended exception that the "controlled species" in the locally analogous system cannot transmit any feedback information. In general, for this interpretation to be valid, the breaking point (where a regulatory function is substituted by a constant value) must be upstream of where the perturbation occurs; this can be ensured for all types of perturbations if the feedback is broken right where the feedback link function occurs (as proposed here).

If the system has multiple feedback loops, and hence multiple feedback link functions need to be defined, we can either evaluate the contribution of each one individually or any combination of them. In either case, the process proceeds exactly as detailed above.

It is essential that the original system and its locally analogous system differ only by the absence of the feedback link, such that their differential response can be unequivocally associated with the role of the feedback regulation. Then, when implementing the 4(a) strategy described above, the interaction of the auxiliary species with the downstream elements on the feedback must perfectly mimic that of the controlled species, and the

perturbation being evaluated must not affect the auxiliary species (e.g. changing dilution). When implementing the 4(b) strategy described above, the constant value substituting the feedback link function must take into account all the parameter values involved in the original feedback link function, with the limitation that perturbations on how the controlled species interacts with the rest of the system cannot be evaluated.

5. Calculate the **controlled species steady-state values** for both systems before and after a perturbation of interest, and obtain the associated CoRa value. For the example described above, for each specific parameter set Θ:
   (a) Calculate the controlled species steady state $Y_{ss}|_\Theta$ for the original system.
   (b) Calculate the controlled species steady state $Y_{ss,NF}|_\Theta$ for the locally analogous system without feedback system. Confirm that $Y_{ss} = Y_{ss,NF}$.
   (c) Perturb the desired specific parameter $\rho \in \Theta$ by a small amount, $\rho \rightarrow \rho'$ (e.g. $\mu_Y \rightarrow 1.05 \cdot \mu_Y$).
   (d) Re-calculate the controlled species steady state $Y_{ss}|_{\Theta,\rho\rightarrow\rho'}$ for the original system.
   (e) Re-calculate the controlled species steady state $Y_{ss,NF}|_{\Theta,\rho\rightarrow\rho'}$ for the locally analogous system without feedback.
   (f) Calculate the CoRa value:

$$\text{CoRa}_\Theta(Y,\rho) \quad = \quad \frac{\log\left(\frac{Y_{ss}|_{\Theta,\rho\rightarrow\rho'}}{Y_{ss}|_\Theta}\right)}{\log\left(\frac{Y_{ss,NF}|_{\Theta,\rho\rightarrow\rho'}}{Y_{ss,NF}|_\Theta}\right)}$$

6. Once the system of interest and its locally analogous no-feedback system have been defined, the CoRa analysis can be easily applied over again to **any parameter set and perturbation of interest**:
   (a) Update the specific value of the auxiliary species (e.g. $\mu_{Y*} = \mu_Y W_{ss}$) or the constant parameter (e.g. $\mu_{U*} = \mu_U Y_{ss}$) such that the constant input values in the no-feedback system are identical once again to the feedback link function values in the feedback system (as described on step #4), keeping the no-feedback system locally-analogous to the feedback system before the perturbation to be evaluated occurs.
   (b) Re-calculate the steady-state controlled species responses to the perturbation of interest (step #5).

   Note: Given that CoRa can be calculated for any parameter set of interest, we can easily explore a range of values of specific parameter $\theta \in \Theta$. The resulting *CoRa line* (e.g. Fig. 1D, Fig. 2C-D, and Fig. 3 in the main manuscript) is a representation of the capacity of feedback to mediate adaptation of the system's output to perturbations to the parameter ρ for every value of θ considered. In this work, θ is limited to a change in an individual parameter.

S2.2 Algorithm for CoRa implementation

An algorithm to implement CoRa is available at https://github.com/mgschiavon/CoRa using Julia language. Briefly, the pseudo-code representing this algorithm is presented in *Algorithm 1*.

S3 Analysis of a modified antithetic feedback control strategy using CoRa

We consider a modified *antithetic feedback motif* (ATF; based on Briat *et al.* [2]) with a simple controlled subsystem consisting of a single molecule Y. The ATF motif consists of two molecules U and W that bind to each other forming a transitory complex C. C is then degraded leading to the disappearance of both U and W. Y is produced at a rate that depends on the concentration of W, while U synthesis is induced by Y. The equations of the full system with

Algorithm 1 Calculate CoRa line for a range of conditions

```
Include model equations, md.jl
    odeFB := ODE feedback system
    odeNF := ODE no-feedback system
    localNF := Function to adjust odeNF parameters to make it locally analogous to odeFB
Include model parameter values, par.jl
    Θ := Biochemical parameter values
Include perturbation instructions, pert.jl:
    ρ := Parameter to be perturbed, ρ ∈ Θ
    δ := Perturbation size (Δρ)
    θ := Condition parameter over which to calculate the line, θ ∈ Θ
    r := Range of conditions to evaluate (i.e. θ values to evaluate)
for i ∈ r do
    Θ[θ] ← i                                        ▷ Update θ value.
    Y_ss ← SS(odeFB(Θ))                             ▷ Get controlled species steady state.
    Θ_NF ← localNF(Y_ss, Θ)                         ▷ Get parameters for locally analogous system.
    Y_NF,ss ← SS(odeNF(Θ_NF))                       ▷ Get analogous "controlled" species steady state.
    if Y_ss ≠ Y_NF,ss then                          ▷ Check for numerical errors.
        ERROR: Systems are not locally analogous.
        break
    end if
    Θ[ρ] ← Θ(ρ) ∗ δ                                 ▷ Perturb parameter.
    Θ_NF[ρ] ← Θ_NF(ρ) ∗ δ
    Y_ss,D ← SS(odeFB(Θ))                           ▷ Get controlled species steady state.
    Y_NF,ss,D ← SS(odeNF(Θ_NF))                     ▷ Get analogous "controlled" species steady state.
    CoRa[i] ← log10(Y_ss,D/Y_ss) / log10(Y_NF,ss,D/Y_NF,ss)   ▷ Calculate CoRa value for given conditions.
end for
return CoRa                                         ▷ Return CoRa line for the range of values provided.
```

feedback are then given by:

$$\frac{d}{dt}U = \mu_U Y - (\gamma + \gamma_U)U - \eta_+ UW + (\eta_0 + \gamma_W)C \tag{S3}$$

$$\frac{d}{dt}W = \mu_W - (\gamma + \gamma_W)W - \eta_+ UW + (\eta_0 + \gamma_U)C \tag{S4}$$

$$\frac{d}{dt}C = \eta_+ UW - (\gamma + \eta_0 + \eta_- + \gamma_U + \gamma_W)C \tag{S5}$$

For Y dynamics, two alternative scenarios can be easily foreseen: W can be either inactivated as a transcription factor once it binds U (ATF v1; Fig. 2A),

$$\frac{d}{dt}Y = \mu_Y W - (\gamma + \gamma_Y)Y \tag{S6}$$

or W retains its transcription factor activity until degraded (ATF v2; Fig. 2B),

$$\frac{d}{dt}Y = \mu_Y (W + C) - (\gamma + \gamma_Y)Y \tag{S7}$$

Here all species are subject to loss by dilution (γ), in addition of their own individual degradation rates ($\gamma_\Box$), $\mu_\Box$ represents the synthesis rate for each molecule (either constitutive, μ_W, or dependent of a transcription factor, μ_U and μ_Y), and η_- is the co-degradation rate of U, W in the complex form C; η_+ is the binding rate of U and W (forming the complex C); and η_0 is the spontaneous unbinding rate of these two molecules (dissociating the complex C).

Choosing Y as the controlled species of interest, the corresponding locally analogous system without feedback maintains the same ODE equations (Eqs. S4-S5, and either Eq. S6 or Eq. S7), with the exception of $\frac{dU}{dt}$,

$$\frac{d}{dt}U = \mu_U Y_* - (\gamma + \gamma_U)U - \eta_+ UW + (\eta_0 + \gamma_W)C \tag{S8}$$

where U synthesis rate now depends on a new molecule Y_* with dynamics

$$\frac{d}{dt}Y_* = \mu_{Y_*} - (\gamma + \gamma_{Y_*})Y_* \tag{S9}$$

such that Y_* is constitutively expressed with synthesis μ_{Y*}. If $\gamma_{Y*} = \gamma_Y$, then the steady state value of the locally analogous system without feedback $Y_{ss,NF}$ is equal to the steady state value of the feedback system Y_{ss} if either $\mu_{Y*} = \mu_Y W_{ss}$ or $\mu_{Y*} = \mu_Y (W_{ss} + C_{ss})$, depending on the feedback system being considered (ATF v1 or ATF v2).

In this case, since Y_* in the locally analogous system without feedback does not depend on any other molecule in the system, its concentration will remain constant after any type of perturbation. As mentioned above, this is an important requirement for the mathematically controlled comparison: if a perturbation also affects Y_* value (e.g. experimental perturbations on dilution, γ), the feedback system and the locally analogous system differ in more than just the feedback information, and the CoRa value cannot be interpreted as simply the feedback contribution.

As described by Briat *et al.* [2], assuming there is no dilution ($\gamma = 0$) as well as no individual degradation of U and W (i.e. independent of the complex formation C; $\gamma_U, \gamma_W = 0$), this system (Eqs. S4-S5) is expected to display perfect step disturbance rejection (integral control or perfect adaptation):

$$\frac{d}{dt}U = \mu_U Y - \eta_+ UW + \eta_0 C$$

$$\frac{d}{dt}W = \mu_W - \eta_+ UW + \eta_0 C$$

$$\text{then } \frac{d}{dt}(U - W) = \mu_U Y - \mu_W$$

$$\text{and if } \frac{d}{dt}U_{ss} = \frac{d}{dt}W_{ss} = 0 \text{ then } Y_{ss} = \frac{\mu_W}{\mu_U} \tag{S10}$$

In other words, Y_{ss} is controlled to a reference value $\frac{\mu_W}{\mu_U}$, to which it returns exactly after any step perturbation to the system, provided that the steady-state exists and it is stable (see Olsman *et al.* [7] for further discussion). This conclusion is independent of the particular subsystem being controlled, W being inactive (Eq. S6) or active (Eq. S7) in the complex form, as well the active degradation rate (η_-), and complex formation dynamics ($\frac{d}{dt}C$).

S3.1 Understanding effect of saturation on modified antithetic feedback control

S3.1.1 ATF control limits with inactive complex

In this section we prove that for the system described in Eqs. S3-S6, if $(\gamma + \gamma_W) > 0$, as Y-synthesis rate (μ_Y) value decreases, $\text{CoRa}_\Theta(Y, \mu_Y) \to 1$. Similarly, if $(\gamma + \gamma_U) > 0$, as μ_Y increases, CoRa saturates with $\text{CoRa}_\Theta(Y, \mu_Y) \to 0.5$. These analytically argued results were corroborated by computational demonstrations.

Proposition 1. *For the system described in Eqs. S3-S6, as $\mu_Y \to \mu'_Y$, $\Delta\log(Y_{ss}) = \Delta\log(\mu_Y) + \Delta\log(W_{ss})$. Here, for brevity, we denote $Y_{ss}|_{\Theta,\mu_Y}$ by Y_{ss}, and $Y_{ss}|_{\Theta,\mu'_Y}$ by Y'_{ss}, and similarly for W_{ss}. Therefore $\Delta\log(Y_{ss}) = \log(Y'_{ss}) - \log(Y_{ss})$, $\Delta\log(W_{ss}) = \log(W'_{ss}) - \log(W_{ss})$, and $\Delta\log(\mu_Y) = \log(\mu'_Y) - \log(\mu_Y)$.*

Proof. Given Eq. S6, the controlled species steady state for the system is

$$Y_{ss} = \Big(\frac{\mu_Y}{\gamma + \gamma_Y}\Big)W_{ss} \tag{S11}$$

After a perturbation $\mu_Y \to \mu'_Y$, the new controlled species steady state can be written as

$$Y'_{ss} = \Big(\frac{\mu'_Y}{\gamma + \gamma_Y}\Big)W'_{ss} \tag{S12}$$

Then, the effect of the perturbation on the system can be quantified as

$$\begin{aligned} \Delta\log(Y_{ss}) &= \log(Y'_{ss}) - \log(Y_{ss}) = \log\Big(\frac{Y'_{ss}}{Y_{ss}}\Big) \\ &= \log\Big(\frac{\big(\frac{\mu'_Y}{\gamma+\gamma_Y}\big)W'_{ss}}{\big(\frac{\mu_Y}{\gamma+\gamma_Y}\big)W_{ss}}\Big) = \log\Big(\Big(\frac{\mu'_Y}{\mu_Y}\Big)\Big(\frac{W'_{ss}}{W_{ss}}\Big)\Big) \\ &= \Delta\log(\mu_Y) + \Delta\log(W_{ss}) \end{aligned} \tag{S13}$$

where the effect of the feedback is introduced by the $\Delta\log(W_{ss})$ component.

Consequence 1. In the absence of feedback (i.e. when U and the W do not depend on Y), W_{ss} should remain constant after a μ_Y-perturbation, i.e. $\Delta\log(W_{ss}) = 0$. Then, for this system, the effect of the step μ_Y perturbation is simply equal to the size of the perturbation, i.e. $\Delta\log(Y_{ss}) = \Delta\log(\mu_Y)$.

Consequence 2. By definition, a system has feedback control if the presence of feedback reduces the effect of the perturbation over the controlled species change, i.e. $|\Delta\log(Y_{ss})| < |\Delta\log(\mu_Y)|$. Then, in order to have feedback control, $\Delta\log(W_{ss}) < 0$ if $\Delta\log(\mu_Y) > 0$ (and vice versa). It follows that in a range of μ_Y values with effective feedback control, W_{ss} must decrease monotonically as μ_Y value increases.

Proposition 2. *For the system described in Eqs. S3-S6, if $(\gamma + \gamma_W) > 0$, the total W steady state ($W_{T,ss} = W_{ss} + C_{ss}$) has an upper limit and lower limit that is independent of μ_Y. Additionally, $W_{T,ss}$ approaches its upper limit when $W_{ss} \approx W_{T,ss}$, and its lower limit when $C_{ss} \approx W_{T,ss}$.*

Proof. Let's define total W as the sum of free molecule W and the complex molecule C, i.e. $W_T = W + C$. Then, the equation of change of W_T corresponds to the sum of Eq. S4 and Eq. S5:

$$\begin{aligned} \frac{d}{dt}W_T &= \frac{d}{dt}W + \frac{d}{dt}C \\ &= \mu_W - (\gamma + \gamma_W)(W + C) - \eta_- C \end{aligned} \tag{S14}$$

Without loss of generality, we represent C as a fraction of the total W, αW_T with $\alpha \in [0, 1]$:

$$\frac{d}{dt}W_T = \mu_W - (\gamma + \gamma_W + \alpha\eta_-)W_T \tag{S15}$$

Then, in steady state:

$$W_{T,ss} = \frac{\mu_W}{\gamma + \gamma_W + \alpha\eta_-} \tag{S16}$$

Given that all involved parameters are non-negative, and $\alpha \in [0, 1]$:

$$\begin{aligned} \frac{\mu_W}{\gamma + \gamma_W + \eta_-} \leq & \quad \frac{\mu_W}{\gamma+\gamma_W+\alpha\eta_-} & \leq \frac{\mu_W}{\gamma + \gamma_W} \\ \frac{\mu_W}{\gamma + \gamma_W + \eta_-} \leq & \quad W_{T,ss} & \leq \frac{\mu_W}{\gamma + \gamma_W} \end{aligned} \tag{S17}$$

Notice that the upper limit exists only if $(\gamma + \gamma_W) > 0$. Moreover, it is clear that $W_{T,ss}$ approaches its upper limit when $\alpha \to 0$, i.e. $W_{T,ss} \approx W_{ss}$, while $W_{T,ss}$ approaches its lower limit when $\alpha \to 1$, i.e. $W_{T,ss} \approx C_{ss}$.

Proposition 3. *For the system described in Eqs. S3-S6, and within the range of μ_Y for which the feedback is effective (i.e. $|\Delta\log(Y_{ss})| < |\Delta\log(\mu_Y)|$ for all μ_Y values within the range),* $\text{CoRa}_\Theta(Y, \mu_Y) \to 1$ *as μ_Y decreases, provided that $(\gamma + \gamma_W) > 0$.*

Proof. As $W_{T,ss} = W_{ss} + C_{ss}$ is upper bounded (Eq. S17), W_{ss} must have an upper limit as well (i.e. its supremum, $\sup_{\mu_Y}(W_{ss}) \leq \frac{\mu_W}{\gamma+\gamma_W}$). By *Consequence 2* above, within the μ_Y range where feedback control is effective, W_{ss} value increases as the μ_Y value (before a perturbation is applied) decreases. Therefore, as μ_Y decreases, W_{ss} approaches its supremum, $\sup_{\mu_Y}(W_{ss})$. As this occurs, the increment to its concentration ($\Delta\log(W_{ss})$) after an additional perturbation that decreases the μ_Y value even further (i.e. $\Delta\log(\mu_Y) < 0$) is constrained by the W_{ss} proximity to its limit. With some abuse of notation, we use the symbol $\approx$ to denote the situation in which this limit is taken as W_{ss} approaches its upper bound. As a result, in this regime, $W_{ss} \approx \sup_{\mu_Y}(W_{ss})$ and $\Delta\log(W_{ss}) \approx 0$. Now, using Eq. S13 and *Consequence 1*,

$$\begin{aligned} \text{CoRa}_\Theta(Y, \mu_Y) &= \frac{\Delta\log(\text{Y}_{\text{ss}})}{\Delta\log(\text{Y}_{\text{ss,NF}})} \\ &= \frac{\Delta\log(\mu_{\text{Y}}) + \Delta\log(\text{W}_{\text{ss}})}{\Delta\log(\mu_{\text{Y}})} \\ &\approx \frac{\Delta\log(\mu_{\text{Y}})}{\Delta\log(\mu_{\text{Y}})} && \text{(S18)} \\ &\approx 1 && \text{(S19)} \end{aligned}$$

Proposition 4. *For the system described in Eqs. S3-S6, and within the range of μ_Y for which the feedback is effective (i.e. $|\Delta\log(Y_{ss})| < |\Delta\log(\mu_Y)|$ for all μ_Y values within the range),* $\text{CoRa}_\Theta(Y, \mu_Y) \to 0.5$ *as μ_Y increases, provided that $(\gamma + \gamma_U) > 0$.*

Proof. By *Consequence 2* above, in a range of μ_Y values with feedback control, W_{ss} value decreases as the μ_Y value (before a perturbation is applied) increases. As $W_{T,ss} = W_{ss} + C_{ss}$ is lower bounded (Eq. S17), and $W_{T,ss}$ is minimal when C_{ss} approaches $W_{T,ss}$, C_{ss} must have an lower limit as well (i.e. its infimum, $\inf_{\mu_Y}(C_{ss}) \geq \frac{\mu_W}{\gamma+\gamma_W+\eta_-}$), and $C_{ss} \to \inf_{\mu_Y}(C_{ss})$ as μ_Y increases.

Let's define total U as the sum of free molecule U and the complex molecule C, i.e. $U_T = U + C$. Then, the equation of change of U_T corresponds to the sum of Eq. S3 and Eq. S5:

$$\begin{aligned} \frac{d}{dt}U_T &= \frac{d}{dt}U + \frac{d}{dt}C \\ &= \mu_U Y - (\gamma + \gamma_U)(U + C) - \eta_- C && \text{(S20)} \\ &= \mu_U Y - (\gamma + \gamma_U)U_T - \eta_- C && \text{(S21)} \end{aligned}$$

Let's assume that μ_Y is large enough such that C_{ss} approaches its lower bound, which is given by $c = \inf_{\mu_Y}(C_{ss})$. With some abuse of notation, we use the symbol $\approx$ to denote the situation in which this limit is taken as C_{ss} approaches its lower bound.

$$U_{T,ss} \approx \frac{\mu_U Y_{ss} - \eta_- c}{\gamma + \gamma_U} \tag{S22}$$

and

$$\begin{aligned} U_{ss} &\approx U_{T,ss} - c \\ &= \frac{\mu_U Y_{ss} - (\eta_- + \gamma + \gamma_U)c}{\gamma + \gamma_U} \end{aligned} \tag{S23}$$

Solving Eq. S5 in steady state, and substituting C_{ss}, U_{ss},

$$\begin{aligned} 0 &= \eta_+ U_{ss} W_{ss} - (\gamma + \eta_0 + \eta_- + \gamma_U + \gamma_W) C_{ss} \\ W_{ss} &= \Big(\frac{\gamma + \eta_0 + \eta_- + \gamma_U + \gamma_W}{\eta_+}\Big)\Big(\frac{C_{ss}}{U_{ss}}\Big) \\ &= K_d \Big(\frac{C_{ss}}{U_{ss}}\Big) \\ &\approx K_d \Big(\frac{(\gamma + \gamma_U)c}{\mu_U Y_{ss} - (\eta_- + \gamma + \gamma_U)c}\Big) \end{aligned} \tag{S24}$$

with $K_d := \frac{\gamma+\eta_0+\eta_-+\gamma_U+\gamma_W}{\eta_+}$. Then, solving Eq. S6 in steady state, and substituting W_{ss},

$$\begin{aligned} 0 &= \mu_Y W_{ss} - (\gamma + \gamma_Y) Y_{ss} \\ Y_{ss} &= \frac{\mu_Y}{\gamma + \gamma_Y} W_{ss} \\ Y_{ss} &\approx \Big(\frac{\mu_Y}{\gamma + \gamma_Y}\Big)\Big(\frac{K_d(\gamma + \gamma_U)c}{\mu_U Y_{ss} - (\eta_- + \gamma + \gamma_U)c}\Big) \\ 0 &\approx Y_{ss}^2 - \Big(\frac{(\eta_- + \gamma + \gamma_U)c}{\mu_U}\Big) Y_{ss} - \Big(\frac{\mu_Y K_d(\gamma + \gamma_U)c}{\mu_U(\gamma + \gamma_Y)}\Big) \\ Y_{ss} &\approx \Big(\frac{1}{2}\Big)\Big(\Big(\frac{(\eta_- + \gamma + \gamma_U)c}{\mu_U}\Big) + \sqrt{\Big(\frac{(\eta_- + \gamma + \gamma_U)c}{\mu_U}\Big)^2 + 4\Big(\frac{\mu_Y K_d(\gamma + \gamma_U)c}{\mu_U(\gamma + \gamma_Y)}\Big)}\Big) \\ &= \Big(\frac{(\eta_- + \gamma + \gamma_U)c}{2\mu_U}\Big)\Big(1 + \sqrt{1 + 4\Big(\frac{\mu_Y \mu_U K_d(\gamma + \gamma_U)}{(\gamma + \gamma_Y)(\eta_- + \gamma + \gamma_U)^2 c}\Big)}\Big) \\ &= \Big(\frac{(\eta_- + \gamma + \gamma_U)c}{2\mu_U}\Big)\Big(1 + \sqrt{1 + a \cdot \mu_Y}\Big) \end{aligned} \tag{S25}$$

with $a := 4\Big(\frac{\mu_U K_d(\gamma+\gamma_U)}{(\gamma+\gamma_Y)(\eta_-+\gamma+\gamma_U)^2 c}\Big)$. As a result, the change of the steady-state controlled species Y_{ss} after a small perturbation on μ_Y ($\mu_Y \to \mu_Y'$, used to compute CoRa),

$$\begin{aligned} \Delta\log(Y_{ss}) &= \log\Big(\frac{\big(\frac{(\eta_-+\gamma+\gamma_U)c}{2\mu_U}\big)\big(1 + \sqrt{1 + a \cdot \mu_Y'}\big)}{\big(\frac{(\eta_-+\gamma+\gamma_U)c}{2\mu_U}\big)\big(1 + \sqrt{1 + a \cdot \mu_Y}\big)}\Big) \\ &= \log\Big(\frac{\big(1 + \sqrt{1 + a \cdot \mu_Y'}\big)}{\big(1 + \sqrt{1 + a \cdot \mu_Y}\big)}\Big) \end{aligned} \tag{S26}$$

On the other hand, given *Consequence 1*, the no-feedback system has $\Delta\log(Y_{ss,NF}) = \Delta\log(\mu_Y) = \log(\frac{\mu_Y'}{\mu_Y})$, and the associated CoRa value is given by:

$$\text{CoRa} = \frac{\log\Big(\frac{1+\sqrt{1+a\cdot\mu_Y'}}{1+\sqrt{1+a\cdot\mu_Y}}\Big)}{\log(\frac{\mu_Y'}{\mu_Y})} \tag{S27}$$

As μ_Y increases, with $(a \cdot \mu_Y) \gg 1$, such that $(1 + \sqrt{1 + a \cdot \mu_Y}) \approx \sqrt{a \cdot \mu_Y}$, then

$$\text{CoRa} \approx \frac{\log\Big(\frac{(a\cdot\mu_Y')^{0.5}}{(a\cdot\mu_Y)^{0.5}}\Big)}{\log(\frac{\mu_Y'}{\mu_Y})}$$

$$\approx \frac{0.5\log(\frac{\mu'_Y}{\mu_Y})}{\log(\frac{\mu'_Y}{\mu_Y})}$$

$$\approx 0.5 \quad \text{(S28)}$$

S3.1.2 ATF control limits with active complex

In this section, we demonstrate that for the system described in Eqs. S3-S5,S7, if $(\gamma + \gamma_W) > 0$, as Y-synthesis rate (μ_Y) value decreases, $\text{CoRa}_\Theta(Y, \mu_Y) \to 1$. Similarly, as μ_Y increases, CoRa saturates with $\text{CoRa}_\Theta(Y, \mu_Y) \to 1$, regardless of $\gamma, \gamma_W, \gamma_U = 0$. These analytically argued results were corroborated by computational demonstrations.

Proposition 5. *For the system described on Eqs. S3-S5,S7, as $\mu_Y \to \mu'_Y$, $\Delta\log(Y_{ss}) = \Delta\log(\mu_Y) + \Delta\log(W_{T,ss})$. Here, for brevity, we denote $Y_{ss}|_{\Theta,\mu_Y}$ by Y_{ss}, and $Y_{ss}|_{\Theta,\mu'_Y}$ by Y'_{ss}, and similarly for $W_{T,ss}$. Therefore $\Delta\log(Y_{ss}) = \log(Y'_{ss}) - \log(Y_{ss})$, $\Delta\log(W_{T,ss}) = \log(W'_{T,ss}) - \log(W_{T,ss})$, and $\Delta\log(\mu_Y) = \log(\mu'_Y) - \log(\mu_Y)$.*

Proof. Given Eq. S7, the controlled species steady state for the system is

$$\begin{aligned} Y_{ss} &= \Big(\frac{\mu_Y}{\gamma + \gamma_Y}\Big)(W_{ss} + C_{ss}) \\ &= \Big(\frac{\mu_Y}{\gamma + \gamma_Y}\Big)W_{T,ss} \end{aligned} \quad \text{(S29)}$$

After a perturbation $\mu_Y \to \mu'_Y$, the new controlled species steady state can be written as

$$Y'_{ss} = \Big(\frac{\mu'_Y}{\gamma + \gamma_Y}\Big)W'_{T,ss} \quad \text{(S30)}$$

Then, the effect of the perturbation on the system can be quantified as

$$\begin{aligned} \Delta\log(Y_{ss}) &= \log(Y'_{ss}) - \log(Y_{ss}) = \log\Big(\frac{Y'_{ss}}{Y_{ss}}\Big) \\ &= \log\Big(\frac{\big(\frac{\mu'_Y}{\gamma+\gamma_Y}\big)W'_{T,ss}}{\big(\frac{\mu_Y}{\gamma+\gamma_Y}\big)W_{T,ss}}\Big) = \log\Big(\Big(\frac{\mu'_Y}{\mu_Y}\Big)\Big(\frac{W'_{T,ss}}{W_{T,ss}}\Big)\Big) \\ &= \Delta\log(\mu_Y) + \Delta\log(W_{T,ss}) \end{aligned} \quad \text{(S31)}$$

where the effect of the feedback is introduced by the $\Delta\log(W_{T,ss})$ component.

Consequence 3. In the absence of feedback (i.e. when U and W do not depend on Y), $W_{T,ss}$ should remain constant after a μ_Y-perturbation, i.e. $\Delta\log(W_{T,ss}) = 0$. As a result, the effect of the perturbation on the system is simply equal to the size of the perturbation, i.e. $\Delta\log(Y_{ss}) = \Delta\log(\mu_Y)$.

Consequence 4. By definition, a system has feedback control if the presence of feedback reduces the effect of the perturbation over the controlled species change, i.e. $|\Delta\log(Y_{ss})| < |\Delta\log(\mu_Y)|$. Then, in order to have feedback control, $\Delta\log(W_{T,ss}) < 0$ if $\Delta\log(\mu_Y) > 0$ (and vice versa). It follows that in range of μ_Y values with effective feedback control, $W_{T,ss}$ must decrease monotonically as μ_Y value increases.

Proposition 6. *For the system described in Eqs. S3-S5,S7, if $(\gamma + \gamma_W) > 0$, the total W steady state ($W_{T,ss} = W_{ss} + C_{ss}$) has an upper limit and lower limit, independent of μ_Y. Additionally, $W_{T,ss}$ approaches its upper limit when $W_{ss} \approx W_{T,ss}$, and its lower limit when $C_{ss} \approx W_{T,ss}$.*

Proof. Let's define total W as the sum of free molecule W and the complex molecule C, i.e. $W_T = W + C$. Then, the equation of change of W_T corresponds to the sum of Eq. S4 and Eq. S5:

$$\begin{aligned} \frac{d}{dt}W_T &= \frac{d}{dt}W + \frac{d}{dt}C \\ &= \mu_W - (\gamma + \gamma_W)(W + C) - \eta_- C \end{aligned} \quad \text{(S32)}$$

Without loss of generality, we represent C as a fraction of the total W, αW_T with $\alpha \in [0,1]$:

$$\frac{d}{dt}W_T \quad = \quad \mu_W - (\gamma + \gamma_W + \alpha\eta_-)W_T \tag{S33}$$

Then, at steady state:

$$W_{T,ss} = \frac{\mu_W}{\gamma + \gamma_W + \alpha\eta_-} \tag{S34}$$

Given that all involved parameters are non-negative, and $\alpha \in [0,1]$:

$$\begin{aligned} \frac{\mu_W}{\gamma + \gamma_W + \eta_-} \leq & \quad \frac{\mu_W}{\gamma+\gamma_W+\alpha\eta_-} & \leq \frac{\mu_W}{\gamma + \gamma_W} \\ \frac{\mu_W}{\gamma + \gamma_W + \eta_-} \leq & \quad W_{T,ss} & \leq \frac{\mu_W}{\gamma + \gamma_W} \end{aligned} \tag{S35}$$

Notice that the upper limit exists only if $(\gamma + \gamma_W) > 0$. Moreover, it is clear that $W_{T,ss}$ approaches its upper limit when $\alpha \to 0$, i.e. $W_{T,ss} \approx W_{ss}$, while $W_{T,ss}$ approaches its lower limit when $\alpha \to 1$, i.e. $W_{T,ss} \approx C_{ss}$.

Proposition 7. *For the system described in Eqs. S3-S5,S7 and within the range of μ_Y for which the feedback is effective (i.e. $|\Delta\log(Y_{ss})| < |\Delta\log(\mu_Y)|$), $\mathrm{CoRa}_\Theta(Y,\mu_Y) \to 1$ as μ_Y decreases, provided that $(\gamma+\gamma_W) > 0$.*

Proof. By *Consequence 4*, in the range of effective feedback control, $W_{T,ss}$ value increases as the μ_Y value (before a perturbation is applied) decreases. Therefore, as the μ_Y value decreases, $W_{T,ss}$ approaches its limit, $\frac{\mu_W}{\gamma+\gamma_W}$ (Eq. S35). Therefore, the potential increment to its concentration ($\Delta\log(W_{T,ss})$) after a perturbation that decreases μ_Y value even further (i.e. $\Delta\log(\mu_Y) < 0$) is constrained by the $W_{T,ss}$ proximity to the limit. With some abuse of notation, we use the symbol $\approx$ to denote the situation in which the limit is taken as W_{ss} approaches its upper bound. In this regime, $W_{T,ss} \approx \frac{\mu_W}{\gamma+\gamma_W}$ and $\Delta\log(W_{ss}) \approx 0$. Using Eq. S31 and *Consequence 3*,

$$\begin{aligned} \mathrm{CoRa}_\Theta(Y,\mu_Y) &= \frac{\Delta\log(\mathrm{Y_{ss}})}{\Delta\log(\mathrm{Y_{ss,NF}})} \\ &= \frac{\Delta\log(\mu_\mathrm{Y}) + \Delta\log(\mathrm{W_{T,ss}})}{\Delta\log(\mu_\mathrm{Y})} \\ &\approx \frac{\Delta\log(\mu_\mathrm{Y})}{\Delta\log(\mu_\mathrm{Y})} \\ &\approx 1 \end{aligned} \tag{S36}$$

Proposition 8. *For the system described in Eqs. S3-S5,S7, and within a range in which the feedback is effective (i.e. $|\Delta\log(Y_{ss})| < |\Delta\log(\mu_Y)|$ for all μ_Y values within the range), $\mathrm{CoRa}_\Theta(Y,\mu_Y) \to 1$ as μ_Y increases.*

Proof. By *Consequence 4* above, in a range of μ_Y values with effective feedback control, $W_{T,ss}$ value decreases as the μ_Y value (before a perturbation is applied) increases. Therefore, as the μ_Y value increases, $W_{T,ss}$ approaches its limit, $\frac{\mu_W}{\gamma+\gamma_W+\eta_-}$ (Eq. S35). Then the potential reduction on its concentration ($\Delta\log(W_{T,ss})$) after a perturbation that increases μ_Y value even further (i.e. $\Delta\log(\mu_Y) > 0$) is constrained by the $W_{T,ss}$ proximity to the limit. Then as the μ_Y value (before a perturbation is applied) increases, such that $W_{T,ss} \approx \frac{\mu_W}{\gamma+\gamma_W+\eta_-}$ and $\Delta\log(W_{ss}) \approx 0$ (with the same abuse of notation highlighted above as to limits), using Eq. S31 and *Consequence 3*,

$$\begin{aligned} \mathrm{CoRa}_\Theta(Y,\mu_Y) &= \frac{\Delta\log(\mathrm{Y_{ss}})}{\Delta\log(\mathrm{Y_{ss,NF}})} \\ &= \frac{\Delta\log(\mu_\mathrm{Y}) + \Delta\log(\mathrm{W_{T,ss}})}{\Delta\log(\mu_\mathrm{Y})} \\ &\approx \frac{\Delta\log(\mu_\mathrm{Y})}{\Delta\log(\mu_\mathrm{Y})} \\ &\approx 1 \end{aligned} \tag{S37}$$

Notice this limit exists even if W and U are lost only through their mutual annihilation (i.e. $\gamma, \gamma_W, \gamma_U = 0$), as the active degradation is not spontaneous (i.e. $0 < \eta_- < \infty$).

It must be emphasized that the control limits described above depend directly on the specific subsystem being controlled, and that analytical intuitive expressions might not always be feasible. Nevertheless, CoRa has the advantage of not having to rely on this knowledge.

S4 Negative auto-regulation affecting synthesis represented by Michaelis-Menten function limits control performance in multiple motifs, but is alleviated by ultrasensitivity.

Using CoRa, we efficiently compared four distinct feedback control motifs proposed in the literature [4,5,8,14] (Fig. 3; see Section S5 for for equations and parameter values). For comparison on equal footing, we considered each of these different negative feedback structures controlling the same simple biochemical subsystem. These investigations using CoRa can generate a rich data-set to explore the properties of different molecular implementations associated with the same phenomenological macroscopic function –negative auto-regulation. For example, we observed that the feedback strategies employing repression of synthesis modelled using a standard Michaelis-Menten repression function (Fig. 3B-C) displayed a limit of $\text{CoRa}_\Theta(Y, \mu_Y) \geq 0.5$. This behavior relates to the inevitable saturation of the repression function (see Section S4.1 for an example of an analytical treatment of this limit). A notable exception to this limit occurred for the "brink motif" feedback strategy, a motif that combines antithetic molecular sequestration with an activation-deactivation enzymatic cycle to produce a tuneable ultra-sensitive response [8] (Fig. 3D; see Section S5.4).

These patterns that were computationally pinpointed by the CoRa analysis prompted the hypothesis that adding ultra-sensitivity to motifs with Michaelis-Menten synthesis repression might alleviate the limit of their adaptive behaviors. Using CoRa, we tested this hypothesis by adding a Hill coefficient larger than 1 to the Michaelis-Menten function in different strategies ($f_\square(Y) = \mu_\square \frac{K_D^n}{Y^n + K_D^n}$, where $\mu_\square$ is the maximum synthesis rate, K_D is the EC_{50}, and n is the Hill coefficient). By increasing the system ultrasensitivity with the Hill coefficient, the lower bound of the CoRa line decreased in all cases, indicating improved adaptation capabilities of the control loops. For instance, with $\mu_Y = 1\, min^{-1}$, we get $\text{CoRa}_\Theta(Y, \mu_Y) = \{0.5235, 0.0920, 0.0100\}$ for both the *Negative Feedback + Feed-forward loop* (FFL) model and *Buffering + Negative Feedback* (BNF) model with Hill coefficient $n = \{1, 10, 100\}$, respectively (all other parameters as in Fig. 3B and Fig. 3C, respectively; see Section S6). Furthermore, increasing the ultrasensitivity of the brink motif (BMF) itself by increasing either the Hill coefficient (with $\mu_Y = 1\, min^{-1}$, $\text{CoRa}_\Theta(Y, \mu_Y) = \{0.2996, 0.1113, 0.0240\}$ with Hill coefficient $n = \{1, 10, 100\}$, respectively) or the deactivation rate in its enzymatic cycle [8] (with $\mu_Y = 10\, min^{-1}$, $\text{CoRa}_\Theta(Y, \mu_Y) = \{0.4179, 0.1188, 0.0612\}$ with deactivation rate $\beta_I = \{0.5, 5, 50\}\, nM^{-1}\, min^{-1}$, respectively) improved its ability to adapt (all other parameters as in Fig. 3D; see Section S6). We corroborate that, as shown by Samaniego & Franco [8], the BMF motif displays high ultrasensitivity, and the ultrasensitivity increases as β_I increases. In all cases, higher ultrasensitivity (either by increasing the Hill coefficient n or β_I for BMF) results in improved control performance for some range of μ_Y values ($\text{CoRa}_\Theta(Y, \mu_Y)$ approaching zero). These results strongly suggest that feedback strategies based on Michaelean repression of synthesis are severely limited in their capacity for homeostasis, but can be improved using ultra-sensitive components.

In this case, CoRa was used as a computational hypothesis generator about this general principle, which was then confirmed through further computational and analytical investigations.

S4.1 Understanding effect of saturation on buffering + negative feedback control strategy

System proposed in Hancock *et al.* (2017) Hancock *et al.* (2017) explored a simple model proposed to display perfect adaptation. This system consisted of only two species, one working as a buffer of the other while inhibiting its own synthesis (i.e. negative feedback). The equations of this control strategy with a the simple controlled subsystem used in this paper are:

$$\frac{d}{dt}Y = (\mu_Y - kY) - \beta Y + \beta_P U_P - \gamma_Y Y \tag{S38}$$

$$\frac{d}{dt}U_P = \beta Y - \beta_P U_P - \gamma_{U_P} U_P \quad \text{(S39)}$$

where μ_Y is the maximum synthesis rate of Y, β and β_P are inactivation and activation rates respectively, U_P represents the inactive form of Y, γ_Y and γ_{U_P} are the degradation rates of Y and U_P, respectively, and k is inhibition rate of Y over its own synthesis.

At steady state,

$$U_{P,ss} = \frac{\beta Y_{ss}}{\beta_P + \gamma_{U_P}} \quad \text{(S40)}$$

$$Y_{ss} = \frac{\mu_Y}{k + \beta - \beta \frac{\beta_P}{\beta_P + \gamma_{U_P}} + \gamma_Y} \quad \text{(S41)}$$

Then, assuming $\beta_P \gg \gamma_{U_P}$, Y_{ss} is controlled with a reference value $\frac{\mu_Y}{k+\gamma_Y}$.

We consider a modified implementation of this buffering + negative feedback (BNF) control motif where the feedback has an additional intermediate step:

$$\frac{d}{dt}Y = \mu_Y U - (\gamma + \gamma_Y)Y \quad \text{(S42)}$$

$$\frac{d}{dt}U = f(Y) - (\gamma + \gamma_U)U - \beta U + \beta_P U_P \quad \text{(S43)}$$

$$\frac{d}{dt}U_P = -(\gamma + \gamma_{U_P})U_P + \beta U - \beta_P U_P \quad \text{(S44)}$$

The steady state solution for U and U_P is:

$$U_{ss} = Y_{ss}\Big(\frac{\gamma + \gamma_Y}{\mu_Y}\Big) \quad \text{(S45)}$$

$$U_{P,ss} = \frac{\beta U_{ss}}{\gamma + \gamma_{U_P} + \beta_P} \quad \text{(S46)}$$

For Y, in the case where $f(Y) = \mu_U - kY$ is a linear function:

$$Y_{ss} = \mu_U \frac{\mu_Y}{\mu_Y k + (\gamma + \gamma_U + \beta)(\gamma + \gamma_Y) - \beta(\gamma + \gamma_Y)\frac{\beta_P}{\gamma + \gamma_{U_P} + \beta_P}} \quad \text{(S47)}$$

If we assume that $\gamma + \gamma_{U_P} \approx 0$, then Eq. S47 is reduced to:

$$Y_{ss} = \mu_U \frac{\mu_Y}{\mu_Y k + (\gamma + \gamma_U)(\gamma + \gamma_Y)} \quad \text{(S48)}$$

The system has perfect adaptation only if $\mu_Y k \gg (\gamma + \gamma_U)(\gamma + \gamma_Y)$, in which case the reference value is $\frac{\mu_U}{k}$.

In the case where $f(Y) = \mu_U \frac{K_D}{K_D + Y}$ is a Michaelis-Menten function, steady state solution for Y is:

$$\begin{aligned} Y_{ss} &= \frac{-K_D + \sqrt{K_D^2 + 4K_D\Big(\frac{\mu_Y}{\gamma+\gamma_Y}\Big)\Big(\frac{\mu_U}{\gamma+\gamma_U}\Big)\Big(\frac{\gamma+\gamma_U+\beta_P}{\beta+\gamma+\gamma_U+\beta_P}\Big)}}{2} \\ &= \Big(\frac{K_D}{2}\Big)(-1 + \sqrt{1 + a \cdot \mu_Y}) \end{aligned} \quad \text{(S49)}$$

with $a := \Big(\frac{4}{K_D}\Big)\Big(\frac{1}{\gamma+\gamma_Y}\Big)\Big(\frac{\mu_U}{\gamma+\gamma_U}\Big)\Big(\frac{\gamma+\gamma_U+\beta_P}{\beta+\gamma+\gamma_U+\beta_P}\Big)$. This steady state expression already suggests that perturbations to μ_Y cannot be perfectly controlled anymore. Moreover, we show below that regardless of the parameter values, BNF with a Michaelis-Menten function describing the negative regulation has $\text{CoRa}_\Theta(Y, \mu_Y) > 0.5$.

The corresponding locally analogous system without feedback maintains the same ODE equations (Eq. S42 and Eq. S44), with the exception of $\frac{dU}{dt}$,

$$\frac{d}{dt}U = f(Y_*) - (\gamma + \gamma_U)U - \beta U + \beta_P U_P \quad \text{(S50)}$$

where U synthesis rate now depends on a new molecule Y_* with dynamics

$$\frac{d}{dt}Y_* \;=\; \mu_{Y*} - (\gamma + \gamma_{Y*})Y_* \tag{S51}$$

such that, for each parameter set Θ, Y_* is constitutively expressed with synthesis μ_{Y*} equal to Y synthesis rate in the pre-perturbation steady state solution (i.e. $\mu_{Y*} = \mu_Y U$), and degradation rate $\gamma_{Y*} = \gamma_Y$. Then, with $f(Y_*) = \mu_Y \frac{K_D}{K_D + Y_*}$, the controlled species steady state solution $Y_{ss,NF}$ for this locally analogous system without feedback is:

$$\begin{aligned} Y_{ss,NF} &= \Big(\frac{K_D}{(\frac{\mu_{Y*}}{\gamma+\gamma_{Y*}}) + K_D}\Big)\Big(\frac{\mu_Y}{\gamma+\gamma_Y}\Big)\Big(\frac{\mu_U}{\gamma+\gamma_U}\Big)\Big(\frac{\gamma+\gamma_U+\beta_P}{\beta+\gamma+\gamma_U+\beta_P}\Big) \\ &= \Big(\frac{K_D(\gamma+\gamma_{Y*})}{\mu_{Y*}+K_D(\gamma+\gamma_{Y*})}\Big)\Big(\frac{K_D}{4}\Big)\cdot a \cdot \mu_Y \end{aligned} \tag{S52}$$

Control limits Using Eq. S49 and Eq. S52, the CoRa value for a small perturbation on μ_Y ($\mu_Y \rightarrow \mu_Y'$) is calculated as,

$$\begin{aligned} \text{CoRa}_\Theta(Y,\mu_Y) &= \frac{\log\Big(\frac{(\frac{K_D}{2})(-1+\sqrt{1+a\cdot\mu_Y'})}{(\frac{K_D}{2})(-1+\sqrt{1+a\cdot\mu_Y})}\Big)}{\log\Big(\frac{(\frac{K_D(\gamma+\gamma_{Y*})}{\mu_{Y*}+K_D(\gamma+\gamma_{Y*})})(\frac{K_D}{4})a\cdot\mu_Y'}{(\frac{K_D(\gamma+\gamma_{Y*})}{\mu_{Y*}+K_D(\gamma+\gamma_{Y*})})(\frac{K_D}{4})a\cdot\mu_Y}\Big)} \\ &= \frac{\log\Big(\frac{-1+\sqrt{1+a\cdot\mu_Y'}}{-1+\sqrt{1+a\cdot\mu_Y}}\Big)}{\log(\frac{\mu_Y'}{\mu_Y})} \end{aligned} \tag{S53}$$

First, we show that $\text{CoRa}_\Theta(Y,\mu_Y)$ decreases monotonically as the μ_Y value (before the perturbation) increases (i.e. $d\text{CoRa}_\Theta(Y,\mu_Y)/d\mu_Y < 0$). In order to evaluate the derivative of CoRa, we first need to derive the continuous form of the CoRa function (CoRa^C), which corresponds to CoRa evaluated in the limit as the perturbation size ($\Delta\mu_Y$, with $\mu_Y' = \mu_Y + \Delta\mu_Y$) approaches zero,

$$\begin{aligned} \text{CoRa}^C_\Theta(Y,\mu_Y) &= \lim_{\Delta\mu_Y\rightarrow 0}(\text{CoRa}_\Theta(Y,\mu_Y)) \\ &= \frac{\log\Big(\frac{-1+\sqrt{1+a\cdot(\mu_Y+\Delta\mu_Y)}}{-1+\sqrt{1+a\cdot\mu_Y}}\Big)}{\log(\frac{(\mu_Y+\Delta\mu_Y)}{\mu_Y})}\Big|_{\lim_{\Delta\mu_Y\rightarrow 0}} \\ &= \frac{\log(-1+\sqrt{1+a\cdot(\mu_Y+\Delta\mu_Y)}) - \log(-1+\sqrt{1+a\cdot\mu_Y})}{\log((\mu_Y+\Delta\mu_Y)) - \log(\mu_Y)}\Big|_{\lim_{\Delta\mu_Y\rightarrow 0}} \\ &= \frac{\frac{\log(-1+\sqrt{1+a\cdot(\mu_Y+\Delta\mu_Y)})-\log(-1+\sqrt{1+a\cdot\mu_Y})}{\Delta\mu_Y}}{\frac{\log((\mu_Y+\Delta\mu_Y))-\log(\mu_Y)}{\Delta\mu_Y}}\Big|_{\lim_{\Delta\mu_Y\rightarrow 0}} \\ &= \frac{\frac{d}{d\mu_Y}\log(-1+\sqrt{1+a\cdot\mu_Y})}{\frac{d}{d\mu_Y}\log(\mu_Y)} \\ &= \frac{1}{2}\Big(1+\frac{1}{\sqrt{1+a\cdot\mu_Y}}\Big) \end{aligned} \tag{S54}$$

Then,

$$\begin{aligned} \frac{d}{d\mu_Y}\text{CoRa}^C_\Theta(Y,\mu_Y) &= \frac{d}{d\mu_Y}\Big(\frac{1}{2}\Big(1+\frac{1}{\sqrt{1+a\cdot\mu_Y}}\Big) \\ &= -\frac{a}{4(1+a\cdot\mu_Y)^{\frac{3}{2}}} < 0 \end{aligned} \tag{S55}$$

As all parameters are positive (i.e. $a > 0$ and $\mu_Y > 0$), this derivative is always negative.

From Eq. S53, it is easy to see that as the μ_Y value (before the perturbation) increases, with $(a \cdot \mu_Y) \gg 1$, such that $(-1 + \sqrt{1 + a \cdot \mu_Y}) \approx \sqrt{a \cdot \mu_Y}$, then

$$\begin{aligned} \text{CoRa}_\Theta(Y, \mu_Y) &\approx \frac{\log\Big(\frac{(a \cdot \mu_Y')^{0.5}}{(a \cdot \mu_Y)^{0.5}}\Big)}{\log(\frac{\mu_Y'}{\mu_Y})} \\ &\approx \frac{0.5 \log(\frac{\mu_Y'}{\mu_Y})}{\log(\frac{\mu_Y'}{\mu_Y})} \\ &\approx 0.5 \end{aligned} \tag{S56}$$

It follows that regardless of the parameter values, BNF with a Michaelis-Menten function describing the negative synthesis regulation has $\text{CoRa}_\Theta(Y, \mu_Y) > 0.5$.

S4.2 *Feedback effectiveness* metric, control saturation and ultrasensitivity

The *feedback effectiveness* metric (FE) proposed by Savageau [10] (see Section S1) also allows for a structural explanation for the observation described above. In the work in [10], they consider a general end-product feedback regulatory system, represented with a system of differential equations in the S-system form

$$\frac{d}{dt} X_i = \alpha_i \Pi_{j=0}^m X_j^{g_{i,j}} - \beta_i \Pi_{j=0}^m X_j^{h_{i,j}} \tag{S57}$$

where $g_{i,j}, h_{i,j}$ are the "apparent kinetic orders" of the rate terms of species X_j with respect to the species X_i, and $\alpha_i, \beta_i \in \mathbb{R}_{0+}$. They show that for this system $FE = -g_{1,m}\frac{\Pi g}{\Pi h}$, where $g_{1,m}$ represents the apparent kinetic order of the regulatory effect of the "last" species (i.e. output, X_m) over the "first" species (i.e.input, X_1) in the regulatory feedback loop, and $\Pi g, \Pi h$ are the product of all the other apparent kinetic order that compose the cascade in the feedback regulatory loop for the positive and negative effector species, respectively (see [10] for full derivation). In the case of a feedback control system, the feedback loop must be negative, and then $g_{1,m}\frac{\Pi g}{\Pi h} < 0$ and $FE > 0$, corresponding to $\text{CoRa} = \frac{1}{1+FE} < 1$ as expected (see Section S2). In the simple case that all apparent kinetic order of the regulatory effectors are equal to one (i.e. first order mechanisms) except for a Michaelis-Menten repression function (e.g. $0 > g_{1,m} \geq -1$), $0 < FE \leq 1$, resulting in $1 > \text{CoRa} \geq 0.5$. If an ultrasensitive repression function is considered instead (i.e. with a Hill coefficient $n > 1$), FE scales with the ultrasensitivity level, reducing then the lower limit expected for the respective CoRa value. For example, with $g_{1,m} \leq -2$, $FE \geq 2$ and $\text{CoRa} \leq \frac{1}{3}$. Noteworthy, the apparent kinetic order will depend both of the associated Hill coefficient and the value of the EC_{50} parameter relative to the steady state concentration of the effector species. This is in clear agreement with the limit observed for the control efficiency of the FFL and BNF systems (CoRa ≥ 0.5; Fig. 3B and Fig. 3C) with a Michaelis-Menten repressive function, which is then alleviated by the addition of ultrasensitivity.

S5 Comparing Feedback Control Motifs with CoRa

For all systems below, Y represents the controlled species of interest.

S5.1 Feedback by Active Degradation + Positive Feedback with inactive complex

We consider the feedback by active degradation motif with the addition of a positive feedback (FDP; Fig. 3A; [3,14]), i.e. W induces its own synthesis. Here we assume that W retains its transcription factor activity until degraded,

$$\frac{d}{dt} U = \mu_U Y - (\gamma + \gamma_U) U - \eta_+ U W + (\eta_0 + \gamma_W + \eta_-) C \tag{S58}$$

$$\frac{d}{dt} W = \mu_W \Big(\frac{(W + C)}{(W + C) + K_D}\Big) - (\gamma + \gamma_W) W - \eta_+ U W + (\eta_0 + \gamma_U) C \tag{S59}$$

$$\frac{d}{dt}C = \eta_+ UW - (\gamma + \eta_0 + \eta_- + \gamma_U + \gamma_W)C \tag{S60}$$

$$\frac{d}{dt}Y = \mu_Y(W + C) - (\gamma + \gamma_Y)Y \tag{S61}$$

Here all species are subject to loss by dilution (γ), in addition of their own individual degradation rates ($\gamma_\Box$), $\mu_\Box$ represents the synthesis rate for each molecule, K_D is the Michaelis-Menten constant for W auto-regulation, and η_- is the active degradation rate of W in the complex form C; η_+ is the binding rate of U and W (forming the complex C); and η_0 is the spontaneous unbinding rate of these two molecules (dissociating the complex C).

The corresponding locally analogous system without feedback maintains the same ODE equations (Eq. S59-S61), with the exception of $\frac{dU}{dt}$,

$$\frac{d}{dt}U = \mu_U Y_* - (\gamma + \gamma_U)U - \eta_+ UW + (\eta_0 + \gamma_W + \eta_-)C \tag{S62}$$

where U synthesis rate now depends on a new molecule Y_* with dynamics

$$\frac{d}{dt}Y_* = \mu_{Y_*} - (\gamma + \gamma_{Y_*})Y_* \tag{S63}$$

For each parameter set Θ, Y_* is constitutively expressed with synthesis μ_{Y*} equal to Y synthesis rate in the pre-perturbation steady state solution (i.e. $\mu_{Y*} = \mu_Y(W_{ss} + C_{ss})$), and degradation rate $\gamma_{Y*} = \gamma_Y$.

S5.2 Feedback + Feedforward Loop

We consider a motif with negative feedback and a coherent feed-forward loop (FFL; Fig. 3B), similar to the one proposed in Harris *et al.* [5], where Y represses the synthesis of U, and U induces the synthesis of both Y and W, which in turns also induces Y synthesis:

$$\frac{d}{dt}U = \mu_U\Big(\frac{K_D}{Y + K_D}\Big) - (\gamma + \gamma_U)U \tag{S64}$$

$$\frac{d}{dt}W = \mu_W U - (\gamma + \gamma_W)W \tag{S65}$$

$$\frac{d}{dt}Y = \mu_Y(U + W) - (\gamma + \gamma_Y)Y \tag{S66}$$

Here all species are subject to loss by dilution (γ), in addition of their own individual degradation rates ($\gamma_\Box$), and $\mu_\Box$ represents the synthesis rate for each molecule.

The corresponding locally analogous system without feedback maintains the same ODE equations (Eq. S65-S66), with the exception of $\frac{dU}{dt}$,

$$\frac{d}{dt}U = \mu_U\Big(\frac{K_D}{Y_* + K_D}\Big) - (\gamma + \gamma_U)U \tag{S67}$$

where U synthesis rate now depends on a new molecule Y_* with dynamics

$$\frac{d}{dt}Y_* = \mu_{Y_*} - (\gamma + \gamma_{Y_*})Y_* \tag{S68}$$

For each parameter set Θ, Y_* is constitutively expressed with synthesis μ_{Y*} equal to Y synthesis rate in the pre-perturbation steady state solution (i.e. $\mu_{Y*} = \mu_Y(U_{ss} + W_{ss})$), and degradation rate $\gamma_{Y*} = \gamma_Y$.

S5.3 Buffering + Negative Feedback

We consider a motif with negative feedback and a buffering loop (BNF; Fig. 3C), similar to the one proposed in Hancock *et al.* [4], where Y represses the synthesis of U, and U transitions to an alternative state U_P and vice versa:

$$\frac{d}{dt}U = \mu_U\Big(\frac{K_D}{Y + K_D}\Big) - (\gamma + \gamma_U)U - \beta U + \beta_P U_P \tag{S69}$$

$$\frac{d}{dt}U_P = -(\gamma + \gamma_U)U_P + \beta U - \beta_P U_P \tag{S70}$$

closing the feedback with either U_P inducing Y synthesis:

$$\frac{d}{dt}Y = \mu_Y U_P - (\gamma + \gamma_Y)Y \tag{S71}$$

Here all species are subject to loss by dilution (γ), in addition of their own individual degradation rates (γ_Y for Y, and γ_U for both U and U_P), μ_U is the maximum synthesis rate of U (in absence of Y), μ_Y is the synthesis rate of Y (depending on U_P, Eq. S71), and β, β_P are the transition rates from U to U_P, and viceversa.

The corresponding locally analogous system without feedback maintains the same ODE equations (Eq. S70-S71), with the exception of $\frac{dU}{dt}$,

$$\frac{d}{dt}U = \mu_U\Big(\frac{K_D}{Y_* + K_D}\Big) - (\gamma + \gamma_U)U - \beta U + \beta_P U_P \tag{S72}$$

where U synthesis rate now depends on a new molecule Y_* with dynamics

$$\frac{d}{dt}Y_* = \mu_{Y_*} - (\gamma + \gamma_{Y_*})Y_* \tag{S73}$$

For each parameter set Θ, Y_* is constitutively expressed with synthesis μ_{Y*} equal to Y synthesis rate in the pre-perturbation steady state solution (i.e. $\mu_{Y*} = \mu_Y U_{P,ss}$), and degradation rate $\gamma_{Y*} = \gamma_Y$.

S5.4 Brink Motif Feedback

We consider a simple version of the Brink motif (BMF; Fig. 3D) proposed by Samaniego & Franco [8], where A and I bind and annihilate each other (by creating the complex C), A induces the activation of U (U_P to U), while I induces its inactivation (U to U_P), and U induces the synthesis of Y:

$$\frac{d}{dt}C = -\gamma C + \eta_+ AI - \eta_0 C + \beta_A A U_P \tag{S74}$$

$$\frac{d}{dt}U = \mu_U - \gamma U + \beta_A A U_P - \beta_I I U \tag{S75}$$

$$\frac{d}{dt}U_P = -\gamma U_P - \beta_A A U_P + \beta_I I U \tag{S76}$$

$$\frac{d}{dt}Y = \mu_Y U - (\gamma + \gamma_Y)Y \tag{S77}$$

With Y repressing the synthesis of A,

$$\frac{d}{dt}A = \mu_A\Big(\frac{K_D}{Y + K_D}\Big) - \gamma A - \eta_+ AI + \eta_0 C - \beta_A A U_P \tag{S78}$$

$$\frac{d}{dt}I = \mu_I - \gamma B - \eta_+ AI + \eta_0 C - \beta_I I U \tag{S79}$$

Here all species are subject to loss by dilution (γ), $\mu_\square$ represents the synthesis rate for each molecule (except U_P, which is only created by the inactivation of U), η_+ is the binding rate of A and I (forming the complex C), η_0 is the spontaneous unbinding rate of these two molecules (dissociating the complex C); β_A, β_I are the activation and inactivation rates of U, respectively; and K_D is the Michaelis-Menten constant for the transcriptional repression by Y.

The corresponding locally analogous system without feedback maintains the same ODE equations (Eq. S74-S76, and Eq. S79), with the exception of $\frac{dA}{dt}$,

$$\frac{d}{dt}A = \mu_A\Big(\frac{K_D}{Y_* + K_D}\Big) - \gamma A - \eta_+ AI + \eta_0 C - \beta_A A U_P \tag{S80}$$

where A synthesis rate now depends on a new molecule Y_* with dynamics

$$\frac{d}{dt}Y_* = \mu_{Y_*} - (\gamma + \gamma_{Y_*})Y_* \tag{S81}$$

such that Y_* is constitutively expressed with synthesis μ_{Y*} equal to Y synthesis rate in the steady state solution for each parameter set Θ (i.e. $\mu_{Y*} = \mu_Y U_{ss}$), and degradation rate $\gamma_{Y*} = \gamma_Y$, before the perturbation.

S6 Parameter values and models used in the manuscript figures

Fig. 2C

- Model: ATF v1 & v2 (Section S3)
- Parameter values: $\gamma = 1 \times 10^{-4}\, min^{-1}$, $\gamma_U = 1 \times 10^{-4}\, min^{-1}$, $\gamma_W = 1 \times 10^{-4}\, min^{-1}$, $\mu_U = 0.125\, min^{-1}$, $\mu_W = 0.1\, nM\, min^{-1}$, $\eta_0 = 1 \times 10^{-4}\, min^{-1}$, $\eta_+ = 0.0375\, nM^{-1}\, min^{-1}$, $\eta_- = 0.5\, min^{-1}$, $\gamma_Y = 1\, min^{-1}$

Fig. 2D

- Model: ATF v1 & v2 (Section S3)
- Parameter values: $\gamma = 1 \times 10^{-4}\, min^{-1}$, $\gamma_U = 1 \times 10^{-4}\, min^{-1}$, $\gamma_W = 1 \times 10^{-4}\, min^{-1}$, $\mu_U = 0.125\, min^{-1}$, $\mu_W = 0.1\, nM\, min^{-1}$, $\eta_0 = 1 \times 10^{-4}\, min^{-1}$, $\eta_+ = 0.0375\, nM^{-1}\, min^{-1}$, $\mu_Y = 0.125\, min^{-1}$, $\gamma_Y = 1\, min^{-1}$

Fig. 3A

- Model: FDP (Section S5.1)
- Parameter values: $\gamma = 0.01\, min^{-1}$, $\gamma_U = 0.05\, min^{-1}$, $\gamma_W = 1 \times 10^{-4}\, min^{-1}$, $\mu_U = 0.125\, min^{-1}$, $\eta_0 = 1 \times 10^{-4}\, min^{-1}$, $\eta_+ = 0.0375\, nM^{-1}\, min^{-1}$, $\eta_- = 0.5\, min^{-1}$, $K_D = 0.02\, nM$, $\gamma_Y = 0.1\, min^{-1}$, $\mu_W = 0.333\, nM\, min^{-1}$ (black line; $Y \approx 10\, nM$ for $\mu_Y = 1\, min^{-1}$).

Fig. 3B

- Model: FFL (Section S5.2)
- Parameter values: $\gamma = 0.01\, min^{-1}$, $\gamma_U = 1 \times 10^{-4}\, min^{-1}$, $\mu_U = 2\, min^{-1}$, $K_D = 1\, nM$, $\gamma_Y = 0.1\, min^{-1}$, $\beta = 0.0108\, min^{-1}$, $\beta_P = 0.1565\, min^{-1}$ (black line; $Y \approx 10\, nM$ for $\mu_Y = 1\, min^{-1}$).

Fig. 3C

- Model: BNF (Section S5.3)
- Parameter values: $\gamma = 0.01\, min^{-1}$, $\gamma_U = 1 \times 10^{-4}\, min^{-1}$, $\mu_U = 2\, min^{-1}$, $K_D = 1\, nM$, $\gamma_Y = 0.1\, min^{-1}$, $\beta = 0.0108\, min^{-1}$, $\beta_P = 0.1565\, min^{-1}$ (black line; $Y \approx 10\, nM$ for $\mu_Y = 1\, min^{-1}$).

Fig. 3D

- Model: BMF (Section S5.4)
- Parameter values: $\gamma = 0.01\, min^{-1}$, $\mu_U = 0.1\, nM\, min^{-1}$, $\eta_0 = 1 \times 10^{-4}\, min^{-1}$, $\eta_+ = 0.05\, nM^{-1}\, min^{-1}$, $\beta_A = 0.5\, nM^{-1}\, min^{-1}$, $\beta_I = 0.5\, nM^{-1}\, min^{-1}$, $\gamma_Y = 0.1\, min^{-1}$, $\mu_A = 0.372\, nM\, min^{-1}$, $K_D = 1\, nM$, $\mu_I = 0.125\, nM\, min^{-1}$ (black line; $Y \approx 10\, nM$ for $\mu_Y = 1\, min^{-1}$).

References

1. R. Alves and M. A. Savageau. Extending the method of mathematically controlled comparison to include numerical comparisons. *Bioinformatics*, 16(9):786–798, sep 2000.
2. C. Briat, A. Gupta, and M. Khammash. Antithetic Integral Feedback Ensures Robust Perfect Adaptation in Noisy Biomolecular Networks. *Cell Systems*, 2(1):15–26, jan 2016.
3. T. Drengstig, X. Y. Ni, K. Thorsen, I. W. Jolma, and P. Ruoff. Robust Adaptation and Homeostasis by Autocatalysis. *The Journal of Physical Chemistry B*, 116(18):5355–5363, may 2012.

4. E. J. Hancock, J. Ang, A. Papachristodoulou, and G.-B. Stan. The Interplay between Feedback and Buffering in Cellular Homeostasis. *Cell Systems*, 5(5):498–508.e23, nov 2017.

5. A. W. Harris, J. A. Dolan, C. L. Kelly, J. Anderson, and A. Papachristodoulou. Designing Genetic Feedback Controllers. *IEEE Transactions on Biomedical Circuits and Systems*, 9(4):475–484, 2015.

6. J. G. Lomnitz and M. A. Savageau. Strategy Revealing Phenotypic Differences among Synthetic Oscillator Designs. *ACS Synthetic Biology*, 3(9):686–701, Sept. 2014.

7. N. Olsman, A.-A. Baetica, F. Xiao, Y. P. Leong, R. M. Murray, and J. C. Doyle. Hard Limits and Performance Tradeoffs in a Class of Antithetic Integral Feedback Networks. *Cell Systems*, 9(1):49–63.e16, jul 2019.

8. C. C. Samaniego and E. Franco. Ultrasensitive molecular controllers for quasi-integral feedback. *bioRxiv*, pages 1–27, 2018.

9. M. Savageau. *Biochemical Systems Analysis: A Study of Function and Design in Molecular Biology*. Advanced Book Program. Addison-Wesley Publishing Company, Advanced Book Program, 1976.

10. M. A. Savageau. Concepts relating the behavior of biochemical systems to their underlying molecular properties. *Archives of Biochemistry and Biophysics*, 145(2):612–621, Aug. 1971.

11. M. A. Savageau. Parameter Sensitivity as a Criterion for Evaluating and Comparing the Performance of Biochemical Systems. *Nature*, 229(5286):542–544, Feb. 1971.

12. M. A. Savageau and E. O. Voit. Recasting nonlinear differential equations as S-systems: a canonical nonlinear form. *Mathematical Biosciences*, 87(1):83–115, Nov. 1987.

13. M. Valderrama-Gómez and M. A. Savageau. Phenotype-centric modeling for rational metabolic engineering. *Metabolic Engineering*, May 2022.

14. F. Xiao and J. C. Doyle. Robust Perfect Adaptation in Biomolecular Reaction Networks. *bioRxiv*, 2018.
